# Comparison of Immunological Profiles of SARS-CoV-2 Variants in the COVID-19 Pandemic Trends: An Immunoinformatics Approach

**DOI:** 10.3390/antibiotics10050535

**Published:** 2021-05-06

**Authors:** Jenifer Mallavarpu Ambrose, Vishnu Priya Veeraraghavan, Malathi Kullappan, Poongodi Chellapandiyan, Surapaneni Krishna Mohan, Vivek Anand Manivel

**Affiliations:** 1 Department of Research, Panimalar Medical College Hospital & Research Institute, Varadharajapuram, Poonamallee, Chennai 600 123, Tamil Nadu, India; jenifer.research@pmchri.ac.in (J.M.A.); malathi.research@pmchri.ac.in (M.K.); 2 Department of Biochemistry, Saveetha Dental College, Saveetha Institute of Medical and Technical Sciences (SIMATS), Saveetha University, Velappanchavadi, Chennai 600 077, Tamil Nadu, India; vishnupriya@saveetha.com; 3 Department of Obstetrics & Gynaecological Nursing, Panimalar College of Nursing, Varadharajapuram, Poonamallee, Chennai 600 123, Tamil Nadu, India; cpoongodi@pcon.ac.in; 4 Departments of Biochemistry, Molecular Virology, Clinical Skills and Simulation, Panimalar Medical College Hospital & Research Institute, Varadharajapuram, Poonamallee, Chennai 600 123, Tamil Nadu, India; 5 Rudbeck Laboratory, Department of Immunology, Genetics and Pathology, Uppsala University, 75185 Uppsala, Sweden

**Keywords:** SARS-CoV-2 variants, SARS-CoV-2 antigenicity, SARS-CoV-2 immunogenicity, SARS-CoV-2 epitope prediction, immunoinformatics approach, COVID-19 severity, infection rate, epidemiological fitness, vaccine modification

## Abstract

The current dynamics of the COVID-19 pandemic have become a serious concern with the emergence of a series of mutant variants of the SARS-CoV-2 virus. Unlike the previous strain, it is reported that the descendants are associated with increased risk of transmission yet causing less impact in terms of hospital admission, the severity of illness, or mortality. Moreover, the vaccine efficacy is also not believed to vary among the population depending on the variants of the virus and ethnicity. It has been determined that the mutations recorded in the spike gene and protein of the newly evolved viruses are specificallyresponsible for this transformation in the behavior of the virus and its disease condition. Hence, this study aimed to compare the immunogenic profiles of the spike protein from the latest variants of the SARS-CoV-2 virus concerning the probability of COVID-19 severity. Genome sequences of the latest SARS-CoV-2 variants were obtained from GISAID and NCBI repositories. The translated protein sequences were run against T-cell and B-cell epitope prediction tools. Subsequently, antigenicity, immunogenicity, allergenicity, toxicity, and conservancy of the identified epitopes were ascertained using various prediction servers. Only the non-allergic and non-toxic potential epitopes were matched for population relevance by using the Human Leucocyte Antigen population registry in IEDB. Finally, the selected epitopes were validated by docking and simulation studies. The evaluated immunological parameters would concurrently reveal the severity of COVID-19, determining the infection rate of the pathogen. Our immunoinformatics approach disclosed that spike protein of the five variants was capable of forming potential T and B-cell epitopes with varying immune responses. Although the Wuhan strain showed a high number of epitope/HLA combinations, relatively less antigenicity and higher immunogenicity results in poor neutralizing capacity, which could be associated with increased disease severity. Our data demonstrate that increased viral antigenicity with moderate to high immunogenicity, and several potential epitope/HLA combinations in England strain, the USA, India, and South Africa variants, could possess a high neutralizing ability. Therefore, our findings reinforce that the newly circulating variants of SARS-CoV-2 might be associated with more infectiousness and less severe disease condition despite their greater viremia, as reported in the recent COVID-19 cases, whichconsequently determine their increased epidemiological fitness.

## 1. Introduction

The recently emerged SARS-Cov-2 is an RNA virus with high mutating efficiency, and the infection is transmissible between humans [1]. It enters with the help of angiotensin-converting enzyme-2 (ACE-2) receptors present in the upper respiratory tract of the host, which are highly expressed in individuals with comorbidities including old age, diabetes, obesity, and high blood pressure [2,3,4]. Further, there are reports regarding the recently evolved variants and strains of SARS-CoV-2 around the world causing more infections with higher transmissible efficiency. At the same time, the availability of prophylactic vaccine has also been helpingto curb the progression of this infectious disease among the public. It is believed that several single nucleotide variations (SNVs) or the point mutations observed in the SARS-CoV-2 genome and proteome have increased the viability of the pathogen to the environmental stress, efficiency of transmission, and subsequently alter the clinical outcomes [5]. In particular, pathogen–host interaction is a complex process where the pathogen is processed by the pattern recognition receptors and presented by antigen-presenting cells to T and B-cells to elicit an effective immune response [6,7]. The immune response as evaluated by detectable IgG antibody levels and cytotoxic T-cell activity depends on the antigen that cancause varying symptoms ranging from mild fever to lethality [8,9,10]. Such an immune response elicited towards the virus is affected by several factors such as the evolution of the virus, ethnicity, and geography [11]. However, the immunological profiles that the antigens of the variant and specific strains of SARS-CoV-2 present to the host immune system remain unclear. Comparing the various mutants of SARS-CoV-2 in light of antigenicity, immunogenicity, epitope/HLA combinations, and ethnicity will hint not only at the efficacy of the existing and the developing vaccines but also the severity of COVID-19 and its infection rate. In other words, we hypothesized that the comparative analysis of epitope/human leukocyte allele (HLA) combinations recognized in each SARS-CoV-2 variant and their immunological parameters would reveal the severity of COVID-19, determining its epidemiological fitness.

Although reverse vaccinology techniques have been conventionally used to design peptide-based vaccines [12], advanced bioinformatics tools can also be leveraged to predict the immunogenicity of the emerging pathogens within a short period. Mutations such as D614G and N501Y [13,14] have been commonly observed in the S protein of the variants reported from several countries as correlating with higher transmissibility rates of 30–70% [15]. Moreover, of all the structural and non-structural proteins in the SARS-CoV-2 proteome, the spike protein is the major antigen inducing protective immune responses and transmission in the hosts. Hence, it is important to closely monitor the antigenic evolution of the spike in the recently emerged and circulating variants [16,17,18]. A concrete understanding of the antigen that the isolates present to the host immune system and their ability to elicit an increased cell-mediated immune response in the human host are lacking. Therefore, this study aimed at identifying and comparing the immunogenic cytotoxic T lymphocyte (CTL), helper T lymphocyte (HTL) epitopes, and B-cell epitopes in five different isolates.

## 2. Results

### 2.1. Analysis of the SARS-CoV-2 Spike Glycoprotein Target Sequences

Since the so-called UK variant with mutations like N501Y, D614G, etc., has been widely reported in many countries from October 2020, four representative sequences of the S protein with the characteristic mutations deposited from England, Karnataka in India, Georgia in the United States, and Western Capein South Africa were retrieved. Among the sequences retrieved, three were genome sequences obtained from GISAID submitted by England, India, and South Africa. The protein sequence of the USA variant was instantly obtained from the NCBI-Protein database. To compare these latest variants, the primary Wuhan SARS-CoV-2 genome and the sequence of S glycoprotein were separately retrieved from the NCBI-Protein database. The spike gene of England, India, and South African variants were translated into protein sequences using the BioEdit-translate program.

All five sequences chosen for the study were 1273 amino acids long. Protparam results for physicochemical properties revealed that the composition of the acidic and basic amino acids in the S protein of England, the USA, India, and South Africa showed a slight variation when compared to the Wuhan reference strain.Due to the high number of basic amino acids in all the isolates, they were found to be acidic. Although the instability index value of all five SARS-CoV-2 isolates was found to be within the stable range, it was slightly increased in the spike protein of the recently evolved variants. Interestingly, the negative GRAVY index values of the spike protein in all the five isolates indicated their hydrophilic nature (Table 1).The conservation of the selected proteins at the majority of the sites in the three recently emerged variants indicated the cross-protection ability of their potential epitope candidates (Supplementary Figure S1). However, as represented in the protein variability test results (Table S1), we suggest that the antigenic and immunogenic properties of the protein might be altered in the latest variants based on those few variations observed in their sequences.

**Figure 1:**
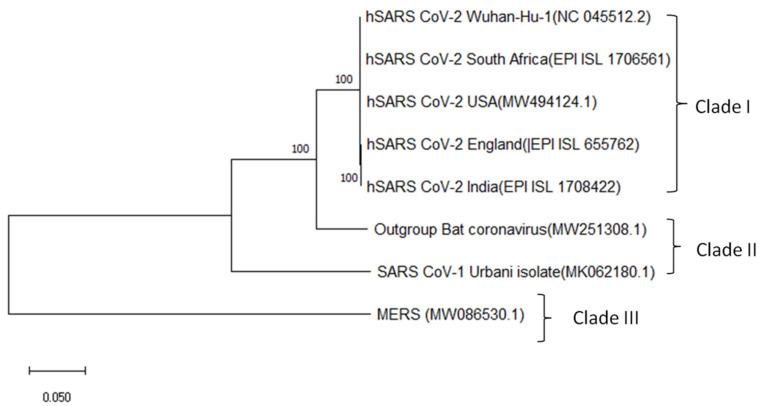
Maximum likelihood phylogenetic tree of SARS-CoV-2 genomes isolated from different geographical locations, namely Wuhan, England, the USA, India, and South Africa with reference to Bat Coronavirus, SARS-CoV-1, and MERS Virus.

To gain insight into their phylogenetic relationships, a maximum likelihood phylogenetic tree was generated for the genome sequences of all the SARS-CoV-2 variants isolated from England (EPI_ISL_655762), India (EPI_ISL_1708422), South Africa (EPI_ISL_1706561), USA (MW494124.1), and China (NC_045512.2) in comparison with Bat Coronavirus (MW251308.1), SARS-CoV-1 (MK062180.1) and MERS virus (MW086530.1). The resulting cladogram revealed that all the SARS-CoV-2 isolates were in clade I while the SARS-CoV-1 strain remained in clade II, indicating close evolutionary relationships shared by the SARS-CoV-2 isolates with each other (Figure 1). It was evident that the MERS virus in clade III shares a common ancestral relationship with SARS-CoV-1, Bat coronavirus, and SARS-CoV-2 respectively.

### 2.2. CTL and HTL Epitope Identification

#### 2.2.1. CTL Epitope Prediction

The identification of cytotoxic and helper T-cell epitopes in the target antigens is one of the key steps not only in the epitope-driven vaccine design process but also crucial for understanding the immunologic profiles of each SARS-CoV-2 variant. Undoubtedly, it is important to identify and compare the immuno-dominant epitopes that are capable of eliciting an immune response in the host and are responsible for the clinical outcome.Thus, T-cell epitopes were computationally identified on the S protein sequences of the SARS-CoV-2 isolates collected from China, England, the United States, India, and South Africa.With the selected threshold value of 1.00, NetCTL 1.2 server initially predicted 167 CD8^+^ unique T-cell epitopes from the S protein sequence of the Wuhan isolate.After evaluating their antigenicity based on the VaxiJen scores, only 77 epitopes were found to be above the threshold value of 0.5. When the qualified 77 antigenic epitopes were further evaluated, only 35 of them possessed immunogenicity scores greater than 0.00. Besides this, the results revealed that 28 of the 77 epitopes were non-allergic and non-toxic peptides in the Wuhan isolate. All the identified peptides were 9mers. The final set of immunogenic, non-allergic, and non-toxic peptides was evaluated for their MHC I binding affinity using the IEDB recommended NetMHCpanEL 4.1 prediction method. With a reference panel of 27 alleles, this prediction step facilitated the selection of good-quality cytotoxic T-cell epitopes. About 20 CTL epitopes with the percentile rank less than or equal to 1.5 for the MHC I allele binding were filtered out, which were further narrowed down to the 11 best CTL epitopes.

Similarly, from the 86 unique CTL epitopes that were identified on the spike protein of the England isolate, we selected 36 epitopes that exhibited increasedantigenicity, 21 of them with positive immunogenicity scores. Based on the binding affinity of the epitopes with the highest number of MHC-I alleles, seven immuno-dominant epitopes of the England variant wereselected as highly antigenic epitopes. They could potentially interact with a set of the class I MHC alleles with high affinity and elicit the desired humoral immune response in the host system. When the latest variant isolated from the USA was analyzed, 159 unique CTL epitopes were initially recognized, which were further narrowed down to 75 qualified antigens from the VaxiJen scores. Out of the 75 epitopes, 38 peptides were either allergic or toxic. Those antigens with positive immunogenicity scores were selected to be highly antigenic peptides that could interact with the MHC I alleles with high binding affinity. Herein, for the 39 peptides that were predicted to be immunogenic, MHCI binding ability was evaluated. The sevenbest epitopes were identified, and their associated alleles were cataloged. Similarly, 67 of the 146 unique CTL epitopes that were initially identified from the Indian isolate were antigenic, and 41 were immunogenic candidates. Twenty-eight out of the 40 epitopes were discovered to be non-allergic and non-toxic peptides. In the end, 14 were qualified to be the best epitopes based on their binding affinity to the MHC I molecules. Lastly, when we analyzed the spike protein of the South African SARS-CoV-2 isolate, it possessed 162 unique CTL epitopes. Of all the variants, the S protein of the South African isolate showed the highest number of 70 antigenic and 37 immunogenic peptides. Finally, 17 out of 28 non-allergic and non-toxic peptides qualified as potential CTL epitope candidates (Supplementary Table S2). Although the number of epitope/HLA combinations was revealed to be higher in the best epitopes of the Wuhan variant, it was carefully observed that only 27% of them were highly antigenic, whereas, in the England variant, 57% of the top-ranked peptides exhibited higher antigenicity. Similarly, 60% of the high-ranking epitopes were revealed to be highly antigenic in the Indian variant. In the South African variant, which encompassed the maximum number of potential CTL epitopes as high as 17, only 50% of them were identified to be highly antigenic. Conversely, 42% of the selected peptides displayed increased antigenicity in the USA isolate. Besides this, variants of England, India, South Africa, and the USA showed a high number of epitopes with positive immunogenicity scores. However, all the predicted epitopes were discovered to be 100% conserved across the variants evaluated.(Table 2). The CTL epitopes ‘ILDITPCSF’ and STQDLFLPF’ of Wuhan isolate, ‘IAIPTNFTI’ and ‘WTAGAAAYY’ of England variant, and the epitopes ‘VVFLHVTYV’, ‘ILDITPCSF, and ‘FTISVTTEI’ of the USA isolate, ‘FTISVTTEI’ and ‘VVFLHVTYV’ of the Indian isolate, and ‘IAIPTNFTI’ and ‘FTISVTTEI’ of the South African variant, showed the highest affinity for about 9 to16 MHC I molecules, including HLA-B*15:01, HLA-A*01:01, HLA-A*02:06, HLA-A*30:02, HLA-A*32:01, HLA-B*35:01, HLA-B*57:01, HLA-B*58:01, HLA-B*44:03, and HLA-B*44:02 in all five SARS-CoV-2 isolates. The HLAs mentioned above denote the acronym HLA, the gene name such as A, B or C, followed by ‘*’, a two digit number that corresponds to antigen specificity, and assigned allele number. It was also observed that ‘WTAGAAAYY’, ‘GVVFLHVTY’, and ‘GAAAYYVGY’ were commonly found CTL epitopes in all three isolates. The epitope ‘ILDITPCSF’ exhibited the highest affinity in Wuhan and USA isolates. Besides this, the epitope ‘IAIPTNFTI’ was recognized to be a common epitope of England and USA variants with the highest affinity for the MHC I molecules. Likewise, ‘VVFLHVTYV’ was noticed to be common between the USA and Indian isolates. Though there were common epitopes exhibited by all three variants, a few of them were distinguished. Particularly, the epitope ‘STQDLFLPF’ was unique to the Wuhan type only. Similarly, ‘QYIKWPWYI’ was unique to England type only. In the same way, the epitopes ‘YQPYRVVVL’ and ‘YSKHTPINL’ were unique to Indian and South African variants only.

#### 2.2.2. HTL Epitope Prediction

We used the IEDB server to determine the binding affinity of the class II MHC human leukocyte antigen alleles with the helper T lymphocytes using human HLA-DR, HLA-DP, and HLA-DQ combinations. Witha reference panel of 29 HLA alleles, we predicted the binding affinity of MHC II allele-associated 15mers for the spike glycoprotein of the three different SARS-CoV-2 variants with the help of the IEDB recommended method. The epitopes identified were ranked based on their percentile rank scores given in the output. The lower the percentile ranked score, the higher the binding affinity of the epitopes for HTL receptors. Only those peptides with the percentile score threshold ≤ 1.5 were considered as having higher binding affinity for MHC II alleles and thus were selected as the best helper T-cell epitopes (Table 3). The IEDB-MHC II prediction tool returned about 300 unique HTL epitopes in the glycoprotein of each of the strains studied within the ≤1.5 percentile rank threshold. However, when analyzed, the results revealed that the HTL peptides identified were overlapping 15mer fragments sharing their core antigenic peptides. Further, a majority of the core HTL peptides recognized were also identified as the best CTL epitope 9mers in the previous step. About 10–20 best HTL epitopes with the percentile rank less than or equal to1.5 in each of the variants studied are tabulated below. VaxiJen scores confirmed that 14 HTL peptides identified in the Wuhan isolatewere antigens, while the variants, on average, displayed eight peptides as the best HTL epitopes. The results showed that the 15mer peptides such as ‘MFVFLVLLPLVSSQC, ‘PYRVVVLSFELLHAP’, and ‘REFVFKNIDGYFKIY’ possessed the highest affinity to the MHC II molecules in all the three variants analyzed. Among the 62 HTL epitopes, 37 peptides exhibited a strong affinity with HLA-DR alleles, including ‘HLA-DRB1*01:01′, ‘HLA-DPA1*03:01/DPB1*04:02, ‘HLA-DRB1*09:01′, ‘HLA-DRB1*13:02′, and ‘HLA-DRB1*15:01′, suggesting that HLA-DR alleles could be the best grooves for the predicted peptides in all the three variants analyzed.

### 2.3. Analysis of Linear and Conformational B-Cell Epitopes

**Figure 2:**
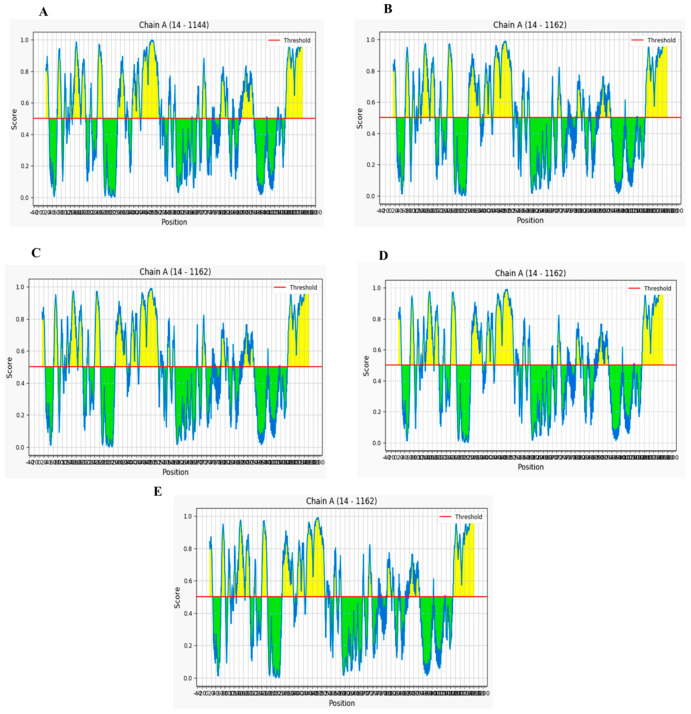
Graphical representation of B-cell linear epitopes of the spike protein of SARS-CoV-2 variants isolated from (**A**) Wuhan, China, (**B**) England, (**C**) USA, (**D**) India, and (**E**) South Africa predicted by Ellipro with a threshold of 0.5, wherein X axis represents sequence position number and Y axis represents Ellipro score. Sequences stretching between the positions 14 and 1162 harbor potential B-cell linear epitopes.

Since the spike glycoprotein of SARS-CoV-2 is structurally oriented on its outer surface, it optimally enhances the specific binding of the pathogen to the ACE II host receptor. For this reason, it was considered an ideal target for B-cell epitope screening. Potential B-cell linear and discontinuous epitopes were identified within the regions they exist in the S protein structure models of China, England, the USA, India, and South Africa using the IEDB-Bepipred linear epitope prediction tool. According to the Bepipred results, out of 35 epitopes predicted, we identified six linear antigenic, non-allergic, and non-toxic epitopes in the S protein of the Wuhan isolate (Supplementary Table S3), which were represented by yellow peaks in the graph, while green-colored slopes indicate the non-epitopic regions within the sequence (Figure 2).Similarly, 8 out of 33 and 6 out of 35 linear epitopes were predicted as the best B-cell epitopes on the S proteins of the England and USA isolates, respectively. Likewise, 7 out of 37 and 9 out of 33 non-allergic and non-toxic peptides were determined to be potential linear epitopes of Indian and South African variants respectively. The predicted epitopes contained regions spanning from 13 to 1269. Overall, out of the 103 linear epitopes predicted in total, only 40 (38%) were antigenic peptides, of which only 18 (17%) were non-allergic and non-toxic epitopes shortlisted from this prediction. It was observed that the epitopes identified within the S protein of the England variant and South African variants were a little morethan those of the other two variants.

Using IEDB—ElliPro, we obtained a total of 88 linear B-cell epitopes with 12, 15, 17, 22, and 22 epitopes identified in China, England, the USA, Indian, and South African variants, respectively. When compared, we noticed that some of the sequential B-cell epitopes predicted in all the five SARS-CoV-2 isolates were similar in terms of length and amino acid composition. The length of the epitopes predicted ranged from 6 to 104. However, certain peptides such as ‘STEKSNIIRGWI’ and ‘TNSPRRARSVA’ were identified in England and USA isolates alone. The conformational epitopes predicted in the Indian and South African variants were noticeably the same. Peptides such as ‘PREGVFVSNGTHWFV’, ‘IAIPTNFTISVTTEI’, ‘SGWTAGAAAYYVG’, ‘VVVLSFELLHAPA’, and ‘SPRRARSVA’ were recognized as overlapping helper T-cell epitopes, which shows the comprehensive immunogenic potential of the epitope candidates against SARS-CoV-2 infection.As far as the B-cell epitopes were concerned, peptides identified as the best epitopes within Wuhan isolate were unique when compared to the recently evolved England and the USA variants, as expected. The peptides predicted in England and the USA variants were the same with the peptide ‘TESNKKFLPFQQF’ unique to the USA isolate only. Interestingly, the B-cell epitopes identified in the Indian and South African isolates exhibited the same patterns, which were different from the England and USA variants (Table 4).

**Figure 3:**
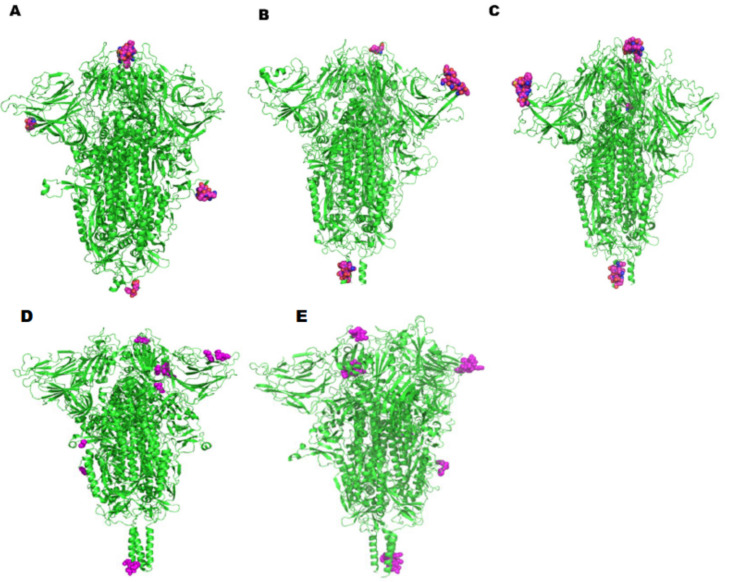
Representations of potential discontinuous B-cell epitopic regions mapped onto the spike protein of the SARS-CoV-2 variants: (**A**) Wuhan, (**B**) England, (**C**) USA, (**D**) India, and (**E**) South Africahighlighted as spheres.

A total of 11, 13, 19, 15, and 26 discontinuous epitopes were calculated at the same exposed surface areas in China, England the USA, Indian, and South African isolates respectively, with a few extra unique epitopes predicted in the mutants, particularly Indian and South African isolates around the same surface regions (Table 5). While most epitopes predicted were exposed on the surface of the spike monomer analyzed, spike_147–154_ (‘KNNKSWME’), spike_496–501_(’GFQPTN’/‘GFQPTY’),and spike_1142–1149_(‘QPELDSFK’) exhibited excellent surface accessibility in the spike trimer. Spike_72–75_(‘GTNG’) was found to be commonly predicted in both England and USA variants, while spike_181–183_(‘GKQ’) was noticed to be unique to the Wuhan isolate only. In parallel, spike_460–462_(‘NLK’) was observed to be unique to the Indian isolate only.However, spike_1158–1161_ (‘NHTS’) peptide was recognized to be a common segment between the Indian and the South African variants. The conformation of these residues was visualized in the Pymol graphics system and highlighted as a sphere (Figure 3). Overall, processing with a combination of B-cell epitope scanning and peptide analysis resulted in the recognition of potential linear and discontinuous epitopes as involved in the epitope formation with slight variation in the Wuhan isolate. Showing distinct variation, England and USA isolates exhibited a few similarities and differences between each other. Surprisingly, Indian andSouth African variants shared close similarities between each other. It is worth mentioning that the top-ranked B-cell epitopes ofall the mutant forms possessed higher antigenicity than that of the Wuhan isolate, similar to the predicted T-cell epitope antigenicity patterns.

### 2.4. Analysis of Population Coverage

HLA composition of a protein varies with diverse ethnic groups and geographical regions aroundthe world. As we focused to compare the epitopes predicted in the five SARS-CoV-2 isolates taken for the study, population coverage was taken into account to evaluate the differences in the candidate epitopes of each variant among diverse populations. In this study, all the potential epitopes identified and their binding MHC I alleles of Wuhan, England, the USA, Indian, and South African isolateswere analyzed for their global population coverage including regions/countries, namely South Asia, India, England, France, Italy, Sweden, United States, and South Africa. The results revealed that all the epitopes exhibited a significant population coverage in different geographic regions of the world, with negligible differences in each of the variants studied.

**Figure 4:**
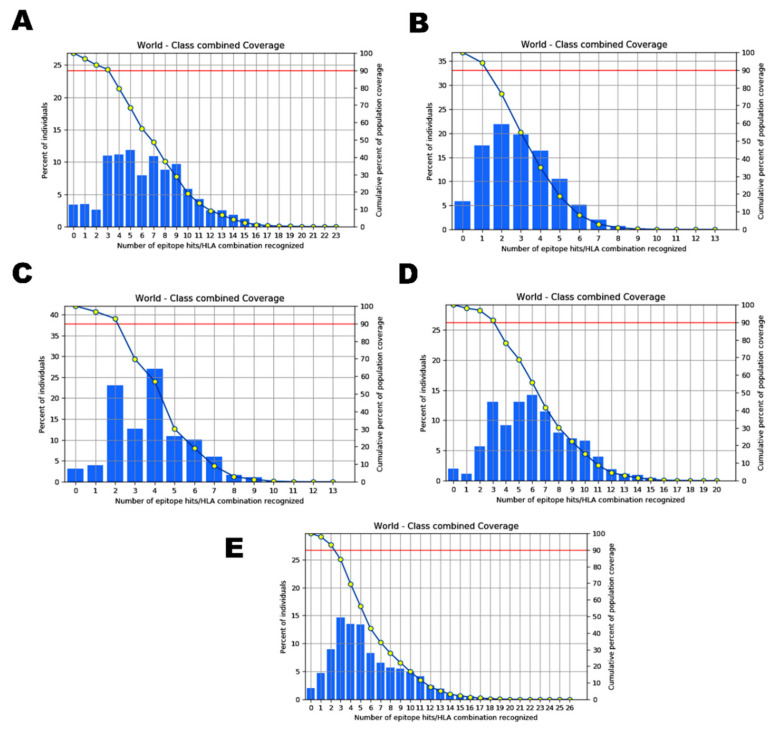
Graphs depicting the world population coverage of the spike glycoprotein of SARS-CoV-2 variants isolated from (**A**) Wuhan, China, (**B**) England, (**C**) the USA, (**D**) India, and (**E**) South Africa to the MHC I and II alleles combined.

The allele distribution of the epitopes identified from the Wuhan isolate covered 93.65% of the global population, while the South Asian population coverage was 88.23% with 80.22% coverage exclusively for the Indian population in particular (Figure 4A). The population coverage for England population was discovered to be 97.08%, and that of other European countries such as France, Italy, and Sweden for the selected alleles was 96.42%, 95.12%,and 92.66% respectively. Similarly, the global population coverage of the alleles that hada binding affinity with the epitopes of the England isolate was 94.15% (Figure 4B). The highest population coverage of 97.62% was observed for the England population as expected. While the South Asian and exclusive Indian population for the same was predicted to be 92.78% and 89.3%, the population of France, Italy, and Sweden showed coverage of 97.36%, 95.84%, and 96.12% respectively. The coverage in the USA population for the alleles of T-cell epitopes identified within Wuhan and England was 93.76%.

In the USA isolate, HLA-alleles of the selected epitopes covered 91.98% of the global population (Figure 4C). The HLA alleles of the Indian isolate covered97.98% of the global population, with an Indian population coverage of 85.34%. The HLA alleles of the Indian isolate showed the maximum coverage for England and France populations (Figure 4D). Similarly, the global population coverage of the alleles binding with the epitopes of the South African variant was close (97.48%) to that of Indian epitope-alleles (Figure 4E). The population coverage patterns between the epitope-alleles of Indian and South African isolates were similar to each other. The highest population coverage, 98.82%, was noticed for the alleles covered in the population of Sweden, which was closely followed by England, France, the United States, and Italy, with population coverage of 99.72%, 99.21%, 98.07%, and 97.97%, respectively. The exclusive Indian population showed remarkably low distribution (85%) for the selected alleles. Furthermore, 95.97% population coverage was predicted for the USA isolate specific allele distribution among England population (Table 6). Among all the five variants, the allele distribution among the Indian and South African populations for the epitopes was exceptionally similar, unlike the rest. While the alleles specific to the epitopes of Wuhan and the USA variants were found to be somewhat similar (87.07% and 83.62%), the same population showed 96.49% for the alleles distribution of the epitopes recognized within the England strain, indicating the diversity of the MHC I and II allele distribution in different ethnic groups (Supplementary Figures S2–S6).

### 2.5. Binding Interactions of the Vaccine Peptides and the HLA Alleles

We generated 14 models from the protein–protein docking between the top-ranked individual epitopes of each variant and their top binding MHC I alleles. The docked complexes generated by PatchDock were further refined using the recommended FireDock server embedded in the PatchDock server. Final models were selected based on the global energy scores, which were computed from individual scores of other interaction parameters such as attractive Vander Waals, repulsive Vander Waals, atomic contact energy, and hydrogen bonding interactions (Table 7). In the Wuhan isolate, peptides ‘GVVFLHVTY ‘(−66.28 kcal/mol) and ‘ILDITPCSF’ (−58.28 kcal/mol) showed a strong binding affinity towards the alleles HLA-B*35:01 and HLA-B*51:01, respectively. We also observed significant interactions between the epitopes ‘WTAGAAAYY’ (−54.98 kcal/mol), ‘GAAAYYVGY’ (−51.90 kcal/mol), ‘VVFLHVTYV’ (−51.05 kcal/mol), and ‘STQDLFLPF’ (−44.18 kcal/mol) with their corresponding alleles HLA-B*35:01, HLA-A*30:02, HLA-B*51:01, and HLA-A*32:01, respectively (Supplementary Figure S7A–D). Among these epitopes, ‘VVFLHVTYV’, ‘WTAGAAAYY’, ‘GAAAYYVGY’, and ‘ILDITPCSF’ were found to be common in the USA isolate. In the England variant, epitopes ‘PYRVVVLSF’ (−65.01 kcal/mol) and ‘QYIKWPWYI’ (−57.28 kcal/mol) exhibited higher affinity with the interacting allele HLA-A*23:01, followed by ‘VYAWNRKRI’ (−46.75 kcal/mol) and ‘NGVEGFNCY’ (−46.33 kcal/mol), which revealed strong binding interactions with the alleles HLA-A*23:01 and HLA-B*35:01, respectively (Supplementary Figure S7E–H). Similarly, the epitopes ‘YQPYRVVVL’, ‘YSKHTPINL’, and ‘FTISVTTEI’ of the Indian variant showed strong binding interactions with the alleles HLA-B*08:01, HLA-A*68:02, and HLA-A*68:02, respectively. Strong binding interactions were observed between the epitopes of South African isolate such as ‘IAIPINFTI’, ‘FTISVTTEI’, and ‘QLTPTWRVY’ and the alleles HLA-B*51:01, HLA-B*51:01, and HLA-B*35:01, respectively (Supplementary Figure S8). We noticed that all the epitopes recognized in the England variant were unique to the specific isolate. Similarly, in the USA isolate, the epitope ‘IAIVMVTIM’ which was unique to the USA variant, possessed strong binding interaction with HLA-B*51:01.

**Figure 5:**
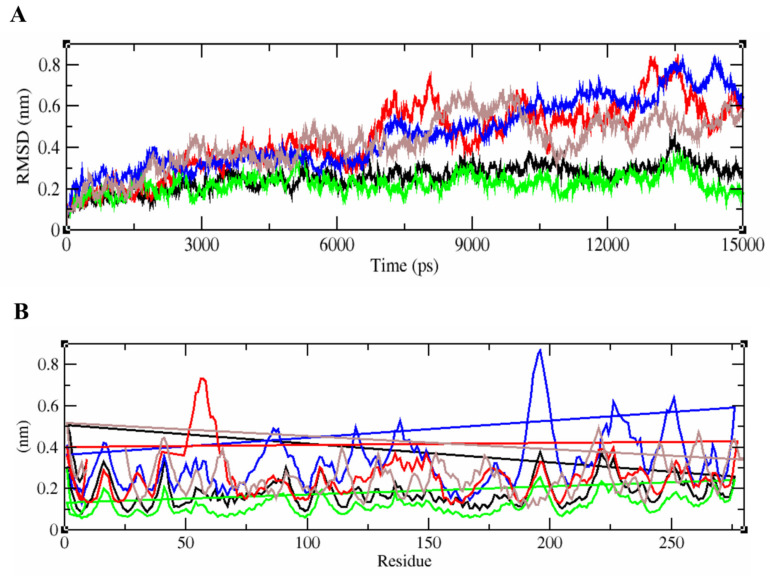
RMSD and RMSF plots generated for the epitope-HLA complexes of Wuhan, England, USA, Indian, and South African variants. (**A**) represents the unstable RMSD values of the complex from England, India, South Africa, USA, and the Wuhan isolates in green, black, brown, red, and blue respectively.The epitope/HLA combinations of England and Indian strains were found to be more stable than that of others. (**B**) represents the fluctuation patterns of the protein–peptide complexes of all five SARS-CoV-2 variants analyzed with their RMSF values given in nm. The amino acid residues of Wuhan strain displayed a maximum deviation in the fluctuation map up to 0.8 nm.

When we subjected the complex to MD simulations and analyzed the physical movements, it was revealed that the tightly bound epitope/HLA complexes of the five SARS-CoV-2 isolates remained stable till 6000 ps. The protein–peptide complex of the Wuhan strain fluctuated and showed the maximum deviation with the RMSD value up to 0.7 nm after 7 ns. complexes with the least binding energies. The side chains of the peptide were deeply buried into the peptide-binding pocket of the macromolecules. Throughout the MD simulation, it was observed that the receptor was stable and the binding antigens of the England and Indian isolates remained attached without any change, unlike the antigen-antibody complex of other isolates. The root-mean-square deviation (RMSD) value determined that the complexes of the England and Indian variants showed the least deviation, unlike those of the other two variants (Figure 5A). Although the docked complex of the USA isolate showed relatively high fluctuation after 6 ns, it was observed to be gradually stabilized at 0.5 nm RSMD. Lastly, the binding conformation of the South African variant displayed moderate deviation between 8000 and 10,000 ps, after which it was consistently stabilized. Similarly, the root-mean-square fluctuation (RMSF) of each residue within the docked complex of the antigen from the England variant was stable, followed by Indian, South African, and USA isolates, respectively. Noticeably, the protein-peptide complex of the Wuhan strain showed relatively more fluctuations with higher RMSF values than the other variants (Figure 5B).

## 3. Discussion

SARS-CoV-2 undergoes rapid evolution leading to the emergence of different lineages and many variants of the pathogen, which have been identified from different parts of the world since its outbreak. Certain mutations reported have been associated with higher transmission rates, complex clinical presentation, and severity of the disease condition, while others didnot. D614G substitution caused by the SNV (A23403G) in the spike protein of a particular SARS-CoV-2 lineage was identified in early February 2020 [13,19]. Another variant, referred to as ‘variant of concern (VOC) 1 December 2020’, showing N501Y mutation in the spike protein and associated with increased infectivity and transmissibility was recorded in South East England initially in December 2020, and the whole of United Kingdom later on. It was discovered that the characteristics of the same N501Y mutation noticed in the British strain were different from that of the South African variant, which evolved simultaneously [19].These signature mutations in the spike protein have caused a resurgence in different geographical regions including India and the USA, which continue to record a high number of COVID-19 cases on daily basis during the second wave of COVID-19. Moreover, newly emerged SARS-CoV-2 variants with the mutated spike gene/protein have been suspected to be moderately or fully resistant to the antibody response elicited by the current generation of COVID-19 vaccines [20]. Although other non-structural proteins such as nsp3, 3CL-pro, and nsp10 have been associated with viral adhering and host invasion, none of them has been experimentally investigated to understand the immunogenicity of the pathogen as well as for the vaccine development [21]. As a spike is a heavily glycosylated protein, investigating the impact of the same on infectivity and immunomodulatory effects is of unquestionable importance [22]. Hence, this study evaluated the immunogenic profiles of the spike protein in the SARS-CoV-2 variants isolated from England, South Africa, the USA, and India in comparison with the Wuhan reference strain.

Our study methodology was based on immunological ‘lock and key theory’, wherein our immuno-genetic makeup is the lock and the SARS-CoV-2 variants are the key [10]. Our approach was independent of comorbidities and therefore assesses the contribution of SARS-CoV-2 variants exclusivelyin terms of antigenicity, immunogenicity, and HLA allele binding affinity makeup. Hence, we have developed a novel method to evaluate the number of potential epitopes with increased antigenicity, immunogenicity, and HLA combinations in the spike protein of the chosen SARS-CoV-2 variants This method has been validated by similar approaches employed for designing T-cell-based peptide vaccine in the past [13]. The immune response against intracellular pathogens can be both humoral and cell-mediated [14,15,23]. For the first time, we have provided a piece of evidence that T-cell immunity is a more major contributor against SARS-CoV-2 than humoral immunity. Although the comparison of S glycoprotein epitopes gives the probability of immunogenic potential and the infection rate of the SARS-CoV-2, we are aware that comparing the whole genome/proteome sequences of the virus might be beneficial in gaining better insight into the varying severity of the variants among different ethnic groups. Apart from predicting immunogenicity, our approach highlights that peptide-based vaccines may prevent side effects by removing toxic peptides found in whole protein [24]. Previous investigations show that recognition of multiple epitopes induces a strong immunogenic response as they increase antibody density [25] and T-cell response [26].

Physicochemical properties predicted for the protein sequences revealed that the mutations in the spike protein of the recently evolved variants have enhanced its structural stability, which in turn promote its efficient binding with the ACE-II receptors as reported previously [27]. Although the conservation of the selected proteins at the majority of the sites in the four recently emerged variants indicated the cross-protection ability of their epitopes, sequence variability at a few sites in those protein sequences, as represented in the protein variability test results, suggests that the antigenic and immunogenic properties of the protein might be altered in the latest variants. It is suggested that these similarities and differences between the spike proteins of all the variants compared might be due to the similarities and differences in the organization of the continuously evolving genome and so the protein-coding control mechanisms [28]. Molecular phylogenetic analysis by the maximum likelihood method traced out the close ancestral relationship shared between all the SARS-CoV-2 variants as they clustered together in the same clade. The orientation of Bat-Coronavirus immediately next to clade I indicated the sufficient divergence existing between them due to the accumulation of single nucleotide mutations. Our results were in agreement with previous reports [29]. Similarly, the relationship between SARS-CoV-1 and SARS-Cov-2 was also evident, as a few genetic variations exist between their genomes with a sequence homology of 85% as mentioned earlier [30].

In the present study, immunoinformatics-based approaches that we exploited predicted potential T-cell epitopes, revealing that certain T-cell epitopes among them were dominantly antigenic, immunogenic, highly conserved, and common in all fiveSARS-CoV-2 isolates studied. However, each variant displayed a few unique epitope candidates as well. In congruence with the previous research findings, a few of the potential epitopes we predicted in our study have been reported in the spike protein of the Wuhan strain [31,32]. Since B lymphocytes are responsible for secreting specific antibodies for neutralizing specific viral particles after invading the host, the full-length sequence of the spike protein of all the three SARS-CoV-2 variants was scanned for putative B-cell epitopes using the experimentally validated data in IEDB using Bepipred linear, Ellipro, and DiscoTope. Our approach has identified overlapping regions of B-cell and T-cell epitopes from the spike protein of the recently evolved SARS-CoV-2 variants, particularly at those sites where those epitopes are 100% identical to the experimentally validated epitopes of the pathogen. This conservation pattern has been observed by other research groups in their epitope prediction study on SARS-CoV-2 proteins [31]. In the spike protein sequences we analyzed, 9mer peptides were mostly recognized by HLA proteins from MHC I, whereas longer epitopes tended to bind MHC class II HLA protein molecules with higher affinities. This observation was in line with the known canonical specifications as reported earlier [31]. Apart from this, population coverage analysis by HLAs exhibited 92 to 97% of the global population for all the predicted T-cell epitopes, with strong binding affinities to MHC I and II, as evidenced from docking and simulation analysis. Furthermore, all the top-scoring T- and B-cell epitopes were predicted to be non-allergic, non-toxic, and of low risk of triggering autoimmune responses, which highlights their immunogenic potential to become vaccine candidates against these latest variants. Immuno-dominant CTL epitopes successfully present themselves depending on their specific binding affinity with MHC I molecules via hydrogen bonds and salt bridge interactions for eliciting antibody response in the host system [33]. After the docked complexes were refined, the interactions between the top-ranked CTL epitopes from each variant and their corresponding MHC I alleles were examined. We noticed multiple hydrogen bond interactions in the complexes. When we analyzed the stability of the protein–protein complex representatives, we found that the potential epitope of the Wuhan strain and its MHC I allele complex was lessstable than that of the other four complexes. On the other hand, the binding confirmations of mutant variants isolated from England, India, South Africa, and the USA were comparatively more stable. From the simulation analysis, it was evident thatmutations in the regions spanning theepitope could bring about a conformational change, which could be responsible for increasing their binding affinity in the formation of rigid complexes.

Overall, the combination of a high number of the best CTL epitopes with several HLA combinations, relatively low antigenicity, and high immunogenicity in the Wuhan spike protein demonstrated its poor neutralization ability. We believe that this reduced neutralization ability could be responsible for increased severity with prolonged symptoms, which in turn might have an impact on the cytokine storms that were previously observed in several COVID-19 cases [34,35]. In contrast, our results showed that the epitopes of England, USA, Indian, and South African variants possessed increased antigenicity, moderate to high immunogenicity, moderate to high T- and B-cell stimulation, and strong host immune system interactions, which in turn might be associated with less severity and mortality rates. However, accumulating mutations in the variants at a high rate could promote their transmissibility rates with increased viremia as evidenced by the latest SARS-CoV-2 reports [36,37]. On the other hand, it is also possible that the new mutants/variants would be able to mitigate this host immune response by evolving HLA-specific mutations like SNVs, cluster-specific reversions, amino acid substitutions, etc. As a result, they could attain the ability to escape the host immune defense, gaining increased severity and resistance against the current vaccines and therapeutic agents [38,39]. Therefore, our data collaterally suggested that the rapid evolution of the surface protein in the SARS-CoV-2 virus has influenced its viral antigenicity, immunogenicity, and epitope/HLA combinations, which could significantly reduce the higher viral load in the clinical manifestations during the recovery process. Furthermore, we suggest that the rapid evolution of the pathogen with the above-mentioned attributes determines an increased epidemiological fitness of the newly evolving SARS-CoV-2 variants [40,41]. Extensive experimental investigations on this might prove to be useful in confirming the biological mechanisms inferred here above.

## 4. Materials and Methods

### 4.1. Collection of Sequence Dataset

We collected the Spike glycoprotein of five different SARS-CoV-2 isolates from five different countries based on the mutations recorded in various geographical regions from the start of the pandemic until January 2021. To begin with, we collected the whole genome sequences of SARS-CoV-2 isolates with N501Y, A570D, D614G, S982A, A262T, E484K, and K417Nmutations from the GISAID repository(https://www.gisaid.org/, accessed on 3 December 2020) deposited from England (accession no EPI_ISL_655762), India (accession no EPI_ISL_1708422), and South Africa (accession no EPI_ISL_1706561) during November and December 2020. The Spike gene sequence was extracted at positions from 21,563 to 25,384 in the genome sequences and translated into S glycoprotein in one of the frames using BioEdit. Since the S glycoprotein of the isolates from Wuhan, China, and the United States deposited in January 2021 were available in NCBI, the protein sequences were directly retrieved from the NCBI-Protein database (https://www.ncbi.nlm.nih.gov/protein/, accessed on 4 January 2021) in FastA format using the accession numbers NC_045512.2 and MW494124.1. Since each isolate that was chosen showed 97 to 100% sequence identity with the sequence of other isolates reported to GISAID during the same timeframe from the same geographical area, this study used a single representative sequence of each variant.

### 4.2. Sequence Variability Analysis of Spike Glycoprotein

To determine the level of the conservancy, all the directly retrieved and translated spike glycoprotein sequences were subjected to variability analysis. A multiple sequence alignment (MSA) was performed for the sequences using the BioEdit-ClustalW multiple alignment program. The absolute site variability in the MSA created was ascertained using Protein Variability Server (PVS) (http://imed.med.ucm.es/PVS/, accessed on 5 January 2021). Among the different variability metrics that were employed by PVS, we identified the conservative fragments in the multiple sequence alignment by plotting the variability. Additionally, the Expasy-Protparam tool (https://web.expasy.org/protparam/, accessed on 5 January 2021) allowed us to qualitatively determine the protein by computing several physicochemical parameters such as molecular weight, theoretical PI, instability index, half-life, aliphatic index, and grand average of hydropathicity (GRAVY) for the selected B-cell epitopes.

### 4.3. Phylogenetic Tree Construction

The evolutionary relationship of five different SARS-CoV-2 isolates from China, England, the United States, India, and South Africa was analyzed using Molecular Evolutionary Genetics Analysis X (MEGA X). Whole genome sequences of the selected isolates were aligned initially via the MEGA-MUSCLE program using the default parameters, and the alignment was exported in MEGA and FastA format. The genome sequences of SARS-CoV-1 (MK062180.1) and Middle-East Respiratory Syndrome virus (MW086530.1) retrieved from the NCBI-Nucleotide database were used as a reference for comparing with the above-mentioned SARS-CoV-2 virus genomes.Whole-genome sequence of Bat coronavirus (MW251308.1) was used as an outgroup for the tree construction [26]. A maximum-likelihood tree was constructed via MEGA X (https://www.megasoftware.net/, accessed on 8 January 2021) with the bootstrap value set to 1000 and Kimura-2 chosen as the tree drawing method.

### 4.4. Prediction of Potential Cytotoxic and Helper T Lymphocyte Epitopes on Spike Glycoprotein of SARS-CoV-2

To understand the CTL and HTL-mediated host immune responses, T-cell epitopes with MHC-I and MHC-II binding affinities were predicted for the highly antigenic S glycoprotein of each variant using NetCTL 1.2 server (https://services.healthtech.dtu.dk/, accessed on 11 January 2021). When the protein sequence was given as input, 9mer CTL epitopes were predicted from the query sequences for all the MHC-I supertypes. This server works based on the training dataset combining the prediction of MHC-I binding peptides, proteasomal C-terminal cleavage, and transporter associated with antigen processing aspects. Only those epitopes with a combined score greater than 1.00 were selected as CTL epitopes. To be stringent in the filtering process, this threshold was adjusted to 1.00, unlike in the previously reported experiments [42]. From the NetCTL output, the antigenicity of unique epitopes was checked for using VaxiJen v2.0 server (http://www.ddg-pharmfac.net/vaxijen/VaxiJen/VaxiJen.html, accessed on 13 January 2020) with 0.5 threshold value, followed by MHC-I binding immunogenicity in Immune Epitope Design Database (IEDB) (http://tools.iedb.org/immunogenicity/, accessed on 15 January 2021). The calculated immunogenicity scores indicates the probability of eliciting an immune response in the host. Epitopes with immunogenicity scores greater than zero were selected as positive epitopes.

MHC-I alleles interacting with each of the unique immunogenic peptides were predicted by the IEDB-MHCI prediction server (http://tools.iedb.org/mhci/, accessed on 18 January 2020). The cut-off value of IC50 was set to less than 200nM [43]. The predicted epitopes were ranked based on lower percentile rank scores, which means the lower the percentile rank score, the higher the binding affinity for the HTL receptors. All those MHC-I alleles with percentile rank less than 1.5 were selected for each unique peptide. Similarly, the helper T lymphocyte epitopes of 15mer length were predicted for all the S protein sequences using IEDB MHC-II binding prediction tool (http://tools.iedb.org/mhcii/, accessed on 20 January 2021), and those alleles with a percentile rank less than 1.5 were selected for further evaluation, as they were considered to be promising alleles with high binding affinity with their corresponding epitopes.

### 4.5. B Lymphocyte Epitope Prediction in SARS-CoV-2 S Protein

With the help of online prediction servers, we forecasted the B cell epitopes on the S glycoprotein sequence of the SARS-CoV-2 variants under study. IEDB-Bepipred Linear Epitope Prediction 2.0 method (http://tools.iedb.org/bcell/, accessed on 23 January 2021), which individually accepted the sequence of the S protein of each variant, was chosen for the prediction of linear epitopes. With 0.5 as the specificity threshold value [35], peptides of varying lengths ranging from 1 to 70 were forecasted as linear epitopes. This prediction method works based on the Random Forest algorithm by correlating the key parameters of the protein such as hydrophilicity, flexibility, surface accessibility, turns, exposed turns, polarity, and propensities of the peptides and thus classifying a particular fragment of the amino acid sequence as potential epitopes. Additionally, we employed IEDB-Ellipro (http://tools.iedb.org/ellipro/, accessed on 25 January 2021) to identify conformational B cell epitopes using the homology-modeled three-dimensional structure of the surface glycoprotein of each variant with a minimum score value of 0.7 and a maximum distance of 6 Å. Ellipro also predicted discontinuous epitopes on the protein sequences submitted. Those epitopes predicted as potential linear epitopes with a high VaxiJen score were selected as the best B-cell linear epitopes. Discontinuous epitopes are increasingly explicit and have more dominant attributes over linear epitopes [25,26]; hence, discontinuous epitopes were additionally forecasted for the S protein of the pathogen isolated from different countries using the IEDB-DiscoTope server. A threshold value of −1.0 was set for selecting epitopes with high specificity and sensitivity. Only those epitopes that fell within the threshold and were oriented on the outer surface of the protein structure were chosen as the best discontinuous B-cell epitopes while discarding the intracellular epitopes above the threshold value.

### 4.6. Prediction of Protective Antigenic Epitopes

For determining the allergenicity of the identified B and T-cell epitopes, AllerTop 1.0 server was used (http://www.ddg-pharmfac.net/allertop/, accessed on 27 January 2021).ToxinPred web server was utilized to examine the toxicity of the identified T-cell epitopes (https://webs.iiitd.edu.in/raghava/toxinpred/index.html, accessed on 27 February 2021). ToxinPred works based on the Support Vector Machine (SVM) algorithm for classifying the toxic and non-toxic peptides depending on their parameters such as mutations, hydropathicity, hydrophilicity, hydrophobicity, and charge.

### 4.7. Conservancy Analysis

The conservation of the identified epitopes across diverse antigens was checked for their conservancy levels using the IEDB-Epitope Conservancy Analysis tool (http://tools.iedb.org/conservancy/, accessed on 28 January 2021) with a sequence identity threshold of 90 percent. The degree of conservancy was determined based on the protein sequence set of SARS-CoV-1 Urbani strain; Wuhan, England, and USA isolates of SARS-CoV-2; and Bat-Coronavirus and reference comprising the epitopes at a specific sequence identity level.

### 4.8. Population Coverage Analysis

Population coverage score was individually calculated against the population of the whole world, South East Asia, United Kingdom, Italy, France, the United States, and South Africa to compare the similarities and differences among those promising T-cell and B-cell epitope candidates across various countries. Due to the difference in the MHC restriction of T-cell response, identifying highly immunogenic peptides from different isolates with diverse HLA binding specificities represents more population coverage in the defined geographical regions. Population coverage for individually qualified T-cell epitope candidates and their binding HLA alleles was assessed by the IEDB Population Coverage Analysis tool (http://tools.iedb.org/population/, accessed on 28 January 2021).

### 4.9. Docking and Simulation

To evaluate the binding affinity between the predicted epitopes selected from each SARS-CoV-2 variant and their corresponding MHC alleles, a molecular docking and simulation study was performed using the in silico tools. For this purpose, the crystal structure of the HLA protein molecules, namely HLA-A*02:06 (3OXR), HLA-B*51:01 (1E27), HLA-B*08:01 (1M05), HLA-A*32:01 (6AT5), HLA-B*57:01 (5VUF), HLA-B*15:01 (6VB3), HLA-A*02:03 (3OX8), HLA-A*01:01(4NQX), HLA-B*35:01 (4LNR), HLA-C*06:02 (5W6A), HLA-A*30:01 (6J1W), HLA-B*07:02 (6AT5), and HLA-A*68:02 (4I48), were retrieved from the RSCB Protein Data Bank (PDB) and prepared for further analysis. For those HLA alleles, namely HLA-A*30:02 and HLA-A*23:01, whose crystal three-dimensional structures were not available in PDB, their molecular sequences were obtained from IMGT/HLA database, and eventually their tertiary structures were modeled using SWISS-MODEL (https://swissmodel.expasy.org/interactive, accessed on 1 February 2021). Similarly, the selected epitopes were modelled using PEP-FOLD server (https://bioserv.rpbs.univ-paris-diderot.fr/services/PEP-FOLD/, accessed on 1 February 2021). Protein–protein docking was performed for the peptide structures and the HLA alleles using PatchDock server (https://bioinfo3d.cs.tau.ac.il/PatchDock/php.php, accessed on 1 February 2021).

Finally, based on their binding energy, for top-scoring protein–protein complexes, molecular dynamics simulation was carried out with GROMACS-2020 (https://manual.gromacs.org/, accessed on 5 February 2021) for 15,000 ps using OPLS force field. We selected TIP3P water model for solvating complexes followed by the addition of ions to neutralize the solvent system. Periodic boundary conditions were set up for orienting the protein–peptide complex. Energy minimization of each system was performed by the steepest descent approach with a tolerance of 1000 kJ/mol/nm. Equilibration of the system was done employingNVT and NPT ensembles with the snapshot interval set to 100 ps. The trajectories were set to be generated every 2 fs and save every 2 ps. All of the trajectories were concatenated to calculate and plot root mean square deviation (RMSD), root mean square fluctuation (RMSF) data, and the protein–protein complexes were analyzed using xmgrace.

## 5. Conclusions

We have attempted to address the raising concerns about the impact of the accumulating mutations in the spike protein of the SARS-CoV-2 variants on their immunogenic potential in driving host–pathogen interactions, the severity of the disease conditions, and the epidemiological fitness by applying the fundamental immunology principles while carrying out in silico simulations.Taken together, there exists a considerable difference between the antigenicity, immunogenicity, and the number of potential epitope/HLA combinations between the newly emerged SARS-CoV-2 variants. Our results corroboratively suggestthat the Wuhan spike protein possesseda higher number of T-cell epitopes with reduced antigenicity and increased immunogenicity, which might lead to increased severity of the disease condition. Nevertheless, the predicted T-cell epitopes disclosed increased antigenicity and moderate to high immunogenic potential with best epitope/HLA combinations in the British, the USA, Indian, and South African strains, making them less virulent and dominantly circulating variant during the current pandemic trends. Therefore, we suggest that the emerging virus strains could be weaker than the original COVID-19 strain, having more transmitting capacity. Alternatively, the emergence of variants could diminish the host immune response by evolving HLA-specific escape mutations, becoming more lethal at a population level. These immunogenic differences that arise among the succeeding variants of SARS-CoV-2 might interfere with the neutralizing activity of the current generation vaccines.

Further, this study highlights that multiple peptides predicted from new variants may be useful to tailor the current vaccines against the latest variants as they have shown worldwide coverage. Nonetheless, further experimental validations are required to confirm the implications of the immunomodulatory effects of the predicted T- and B-cell epitopes among the newly emerged variants before they could be used for controlling the COVID-19 infection caused by the latest descendants of SARS-CoV-2 through the generation of effective immune response and long-term memory with the help ofproperly tailored vaccines.

## Figures and Tables

**Table 1:** Physico-chemical parameters of SARS-CoV-2 isolates studied.

SARS-CoV-2 Isolate	Length	Molecular Weight (Dalton)	Theoretical PI	Total no of −_ve_ and +_ve_ Aminoacids	Extinction Coefficient (M^−1^cm^−1^)	Estimated Half-Life (h)	Instability Index	Aliphatic Index	GRAVY
Wuhan	1273	141,178.47	6.24	110/103	148,960	30	33.01	84.67	−0.079
England	1273	141,169.51	6.32	109/103	150,450	30	33.03	84.67	−0.075
USA	1273	141,120.43	6.32	109/103	148,960	30	32.86	84.67	−0.77
India	1273	141,280.46.	6.35	109/103	150,450	30	32.82	84.45	−0.078
South Africa	1273	141,120.43	6.32	109/103	148,960	30	32.86	84.67	−0.077

**Table 2:** Best cytotoxic T lymphocyte epitope 9mers and their immunogenic characteristics predicted for the three different SARS-CoV-2 variants from the IEDB tool.

SARS-CoV-2 Variant	Epitope	Position	Antigenicity Score	Immunogenicity Score	MHC I Alleles	No of MHC I Binding Alleles	Conservancy at 100% Sequence Identity	Allergenicity	Toxicity
Wuhan, China	ILDITPCSF	584–592	1.184	0.02632	HLA-B*15:01, HLA-A*01:01, HLA-A*02:06, HLA-B*35:01, HLA-B*08:01, HLA-A*02:01, HLA-A*32:01, HLA-A*24:02, HLA-A*23:01, HLA-A*30:02, HLA-B*58:01, HLA-B*53:01	12	100%	Non-allergen	Non-toxic
	STQDLFLPF	50–58	0.662	0.06828	HLA-A*32:01, HLA-B*57:01, HLA-B*15:01, HLA-A*26:01, HLA-B*58:01, HLA-B*35:01, HLA-A*30:02, HLA-A*23:01, HLA-A*24:02, HLA-A*01:01, HLA-A*11:01, HLA-B*53:01	12	100%	Non-allergen	Non-toxic
	VVFLHVTYV	1060–1068	1.512	0.1278	HLA-A*02:06, HLA-A*02:03, HLA-A*02:01, HLA-A*68:02, HLA-B*51:01, HLA-A*30:01, HLA-A*32:01, HLA-B*08:01, HLA-A*26:01	9	100%	Non-allergen	Non-toxic
	GVVFLHVTY	1059–1067	1.410	0.20837	HLA-B*15:01, HLA-A*30:02, HLA-A*26:01, HLA-B*35:01, HLA-A*32:01, HLA-B*57:01, HLA-A*11:01, HLA-A*01:01, HLA-B*58:01	9	100%	Non-allergen	Non-toxic
	WTAGAAAYY	258–266	0.662	0.15259	HLA-A*26:01, HLA-A*01:01, HLA-A*30:02, HLA-A*68:01, HLA-B*35:01, HLA-B*15:01, HLA-B*58:01, HLA-B*57:01	8	100%	Non-allergen	Non-toxic
	GAAAYYVGY	261–269	0.660	0.09963	HLA-A*30:02, HLA-B*15:01, HLA-B*35:01, HLA-A*26:01, HLA-A*01:01, HLA-A*11:01, HLA-B*58:01	7	100%	Non-allergen	Non-toxic
	RVVVLSFEL	509–517	1.192	0.046	HLA-A*32:01, HLA-A*02:06, HLA-B*57:01, HLA-B*58:01, HLA-A*02:01	5	100%	Non-allergen	Non-toxic
England	WTAGAAAY	258–266	0.826	0.15259	HLA-A*26:01, HLA-A*01:01, HLA-A*30:02, HLA-A*68:01, HLA-B*35:01, HLA-B*15:01, HLA-B*58:01, HLA-B*53:01, HLA-B*57:01	9	100%	Non-allergen	Non-toxic
	QYIKWPWYI	1208–1216	1.664	0.21624	HLA-A*24:02, HLA-A*23:01, HLA-C*06:02, HLA-C*07:02, HLA-C*14:02, HLA-A*32:01, HLA-C*07:01	7	100%	Non-allergen	Non-toxic
	GVYFASTEK	89–97	0.664	0.09023	HLA-A*11:01, HLA-A*30:01, HLA-A*68:01, HLA-A*31:01	4	100%	Non-allergen	Non-toxic
	NGVEGFNCY	481–489	1.182	0.22039	HLA-B*35:01, HLA-A*26:01, HLA-C*12:02	3	100%	Non-allergen	Non-toxic
	PYRVVVLSF	507–515	1.028	0.03138	HLA-A*23:01, HLA-A*24:02, HLA-C*14:02	3	100%	Non-allergen	Non-toxic
	VYAWNRKRI	350–358	0.813	0.12625	HLA-A*24:02, HLA-C*14:02, HLA-A*23:01	3	100%	Non-allergen	Non-toxic
	SPRRARSVA	680–688	0.511	0.0402	HLA-B*07:02, HLA-B*08:01	2	100%	Non-allergen	Non-toxic
USA	VVFLHVTYV	1060–1068	1.51	0.1278	HLA-A*02:06, HLA-A*02:03, HLA-A*02:01, HLA-A*68:02, HLA-B*51:01, HLA-A*30:01, HLA-A*30:02, HLA-A*32:01, HLA-B*08:01, HLA-A*26:01, HLA-A*33:01, HLA-A*03:01, HLA-A*31:01, HLA-B*57:01, HLA-B*15:01, HLA-A*68:01	16	100%	Non-allergen	Non-toxic
	ILDITPCSF	584–592	1.184	0.02632	HLA-B*15:01, HLA-A*01:01, HLA-A*02:06, HLA-B*35:01, HLA-B*08:01, HLA-A*02:01, HLA-A*32:01, HLA-A*24:02, HLA-A*23:01, HLA-A*30:02, HLA-B*58:01, HLA-B*53:01	12	100%	Non-allergen	Non-toxic
	GVVFLHVTY	1059–1067	1.140	0.20837	HLA-B*15:01, HLA-A*30:02, HLA-A*26:01, HLA-B*35:01, HLA-A*32:01, HLA-B*57:01, HLA-A*11:01, HLA-B*58:01	8	100%	Non-allergen	Non-toxic
	GAAAYYVGY	1060–1068	0.661	0.09963	HLA-A*30:02, HLA-B*15:01, HLA-B*35:01, HLA-A*26:01, HLA-A*01:01, HLA-A*11:01, HLA-B*58:01	7	100%	Non-allergen	Non-toxic
	WTAGAAAYY	258–266	0.631	0.15259	HLA-A*26:01, HLA-A*01:01, HLA-A*30:02, HLA-A*68:01, HLA-B*35:01, HLA-B*15:01, HLA-B*58:01	7	100%	Non-allergen	Non-toxic
	LPFNDGVYF	84–92	0.559	0.11767	HLA-B*35:01, HLA-B*53:01, HLA-B*51:01, HLA-B*07:02, HLA-A*26:01	5	100%	Non-allergen	Non-toxic
	IAIVMVTIM	1225–1233	1.134	0.06312	HLA-B*51:01, HLA-B*35:01	2	100%	Non-allergen	Non-toxic
India	FTISVTTEI	718–726	0.8535	0.04473	HLA-A*68:02; HLA-A*02:06; HLA-A*02:03; HLA-A*02:01; HLA-B*51:01; HLA-A*26:01; HLA-B*58:01; HLA-A*32:01; HLA-B*53:01	9	100%	Non-allergen	Non-toxic
	VVFLHVTYV	1060–1068	1.512	0.1278	HLA-A*02:06; HLA-A*02:03; HLA-A*02:01; HLA-A*68:02; HLA-B*51:01; HLA-A*30:01; HLA-A*32:01; HLA-B*08:01; HLA-A*26:01	9	100%	Non-allergen	Non-toxic
	YQPYRVVVL	505–513	0.5964	0.1409	HLA-B*08:01; HLA-A*02:06; HLA-B*15:01; HLA-A*02:03; HLA-A*02:01; HLA-A*24:02; HLA-B*40:01; HLA-A*23:01	8	100%	Non-allergen	Non-toxic
	YSKHTPINL	204–212	1.0547	0.9845	HLA-B*57:01; HLA-A*30:01; HLA-B*08:01; HLA-B*58:01; HLA-A*68:02; HLA-B*51:01; HLA-B*15:01; HLA-A*32:01	8	100%	Non-allergen	Non-toxic
	WTAGAAAYY	258–266	0.6306	0.1525	HLA-A*26:01; HLA-A*01:01; HLA-A*30:02; HLA-A*68:01; HLA-B*35:01; HLA-B*15:01; HLA-B*58:01	7	100%	Non-allergen	Non-toxic
	LPFNDGVYF	84–92	0.5593	0.11767	HLA-B*35:01; HLA-B*53:01; HLA-B*51:01; HLA-B*07:02; HLA-A*26:01	5	100%	Non-allergen	Non-toxic
	GAAAYYVGY	261–269	0.6604	0.9963	HLA-A*30:02; HLA-B*15:01; HLA-B*35:01; HLA-A*26:01; HLA-A*01:01	5	100%	Non-allergen	Non-toxic
South Africa	IAIPINFTI	712–720	1.5131	0.27703	HLA-B*51:01; HLA-B*58:01; HLA-B*57:01; HLA-A*02:06; HLA-A*68:02; HLA-B*53:01; HLA-A*32:01; HLA-A*02:01; HLA-A*23:01; HLA-B*35:01; HLA-A*24:02	11	100%	Non-allergen	Non-toxic
	FTISVTTEI	718–726	0.8534	0.04473	HLA-A*68:02; HLA-A*02:06; HLA-A*02:03; HLA-A*02:01; HLA-B*51:01; HLA-A*26:01; HLA-B*58:01; HLA-A*32:01; HLA-B*53:01	9	100%	Non-allergen	Non-toxic
	YQPYRVVVL	505–513	0.5964	0.1409	HLA-B*08:01; HLA-A*02:06; HLA-B*15:01; HLA-A*02:03; HLA-A*02:01; HLA-A*24:02; HLA-B*40:01 HLA-A*23:01	8	100%	Non-allergen	Non-toxic
	WTAGAAAYY	258–266	0.6306	0.15259	HLA-A*26:01; HLA-A*01:01; HLA-A*30:02; HLA-A*68:01; HLA-B*35:01; HLA-B*15:01; HLA-B*58:01	7	100%	Non-allergen	Non-toxic
	YSKHTPINL	204–212	1.0547	0.09845	HLA-B*57:01; HLA-A*30:01; HLA-B*08:01; HLA-B*58:01; HLA-A*68:02; HLA-B*51:01; HLA-A*32:01	7	100%	Non-allergen	Non-toxic
	LPFNDGVYF	84–92	0.5593	0.11767	HLA-B*35:01; HLA-B*53:01; HLA-B*51:01; HLA-B*07:02; HLA-A*26:01	5	100%	Non-allergen	Non-toxic
	GVVFLHVTY	1059–1067	1.4104	0.20837	HLA-B*15:01; HLA-A*30:02; HLA-A*26:01; HLA-B*35:01; HLA-A*32:01	5	100%	Non-allergen	Non-toxic

**Table 3:** List of helper T-cell epitopes with encountering MHC II alleles with their positional, prediction method, antigenicity, allergenicity, and toxicity information.

Sl. No.	Peptide	MHC II Binding Allele	Start	End	Method	Percentile Rank	Vaxijen Score	Allergenicity	Toxicity
Wuhan Isolate									
1	MFVFLVLLPLVSSQC	HLA-DRB1*01:01	1	15	Consensus	0.24	Antigen (0.5741)	Non-allergen	Non-toxic
2	MFVFLVLLPLVSSQC	HLA-DPA1*03:01/DPB1*04:02	1	15	Consensus	0.34	Antigen (0.5741)	Non-allergen	Non-toxic
3	VLLPLVSSQCVNLTT	HLA-DRB4*01:01	6	20	Consensus	1.5	Antigen (0.8957)	Non-allergen	Non-toxic
4	LHSTQDLFLPFFSNV	HLA-DPA1*01:03/DPB1*02:01	48	62	Consensus	1.4	Antigen (0.2110)	Allergen	Non-toxic
5	LFLPFFSNVTWFHAI	HLA-DPA1*01:03/DPB1*04:01	54	68	NetMHCIIpan	0.81	Antigen (0.2477)	Non-allergen	Non-toxic
6	KTQSLLIVNNATNVV	HLA-DRB3*02:02	113	127	NetMHCIIpan	0.17	Antigen (0.6303)	Allergen	Non-toxic
7	SFVIRGDEVRQIAPG	HLA-DRB3*01:01	399	413	Consensus	0.51	Antigen (0.5882)	Non-allergen	Non-toxic
8	GNYNYLYRLFRKSNL	HLA-DRB1*11:01	447	461	Consensus	0.22	Non-antigen (0.1808)	Allergen	Non-toxic
9	PYRVVVLSFELLHAP	HLA-DPA1*03:01/DPB1*04:02	507	521	Consensus	0.25	Antigen (0.8161)	Non-allergen	Non-toxic
10	FNFNGLTGTGVLTES	HLA-DRB1*09:01	541	555	Consensus	0.75	Antigen (0.7797)	Non-allergen	Non-toxic
11	DIPIGAGICASYQTQ	HLA-DQA1*05:01/DQB1*03:01	633	677	Consensus	1.2	Antigen (1.1088)	Non-allergen	Non-toxic
12	IAIPTNFTISVTTEI	HLA-DRB1*07:01	712	726	Consensus	0.47	Antigen (0.7719)	Allergen	Non-toxic
133	CSNLLLQYGSFCTQL	HLA-DRB1*15:01	749	763	Consensus	0.58	Antigen (0.6336)	Non-allergen	Non-toxic
14	WYIWLGFIAGLIAIV	HLA-DQA1*05:01/DQB1*03:01	1214	1228	Consensus	0.58	Antigen (0.5770)	Non-allergen	Non-toxic
15	IWLGFIAGLIAIVMV	HLA-DQA1*05:01/DQB1*03:01	1216	1230	Consensus	0.51	Antigen (0.6150)	Non-allergen	Non-toxic
**England Variant**									
16	FVFLVLLPLVSSQCV	HLA-DRB1*01:01	2	16	Consensus	0.24	Antigen (0.7185)	Non-allergen	Non-toxic
17	KTQSLLIVNNATNVV	HLA-DRB1*13:02	113	127	Consensus	0.01	Antigen (0.6303)	Allergen	Non-toxic
18	YRVVVLSFELLHAPA	HLA-DPA1*01:03/DPB1*04:01	508	522	NetMHCIIpan	0.95	Antigen (0.7072)	Non-allergen	Non-toxic
19	VVLSFELLHAPATVC	HLA-DRB1*01:01	511	525	Consensus	0.03	Antigen (0.8618)	Non-allergen	Non-toxic
20	DIPIGAGICASYQTQ	HLA-DQA1*05:01/DQB1*03:01	663	677	Consensus	1.2	Antigen (1.1088)	Non-allergen	Non-toxic
21	PRRARSVASQSIIAY	HLA-DPA1*02:01/DPB1*14:01	681	695	NetMHCIIpan	1.2	Non-antigen (0.2408)	Non-allergen	Non-toxic
22	YIWLGFIAGLIAIVM	HLA-DQA1*05:01/DQB1*03:01	1215	1229	Consensus	0.51	Antigen (0.6090)	Non-allergen	Non-toxic
**USA Variant**									
23	SSGWTAGAAAYYVGY	HLA-DQA1*05:01/DQB1*03:01	255	269	Consensus	0.94	Antigen (0.6604)	Non-allergen	Non-toxic
24	SGWTAGAAAYYVGYL	HLA-DQA1*05:01/DQB1*03:01	256	270	Consensus	1.2	Antigen (0.6604)	Non-allergen	Non-toxic
25	VVVLSFELLHAPATV	HLA-DPA1*03:01/DPB1*04:02	510	524	Consensus	0.9	Antigen (0.8083)	Non-allergen	Non-toxic
26	DIPIGAGICASYQTQ	HLA-DQA1*05:01/DQB1*03:01	663	677	Consensus	1.2	Antigen (1.1088)	Non-allergen	Non-toxic
27	IAIPTNFTISVTTEI	HLA-DRB1*07:01	712	726	Consensus	0.47	Antigen (0.7719)	Allergen	Non-toxic
28	RSFIEDLLFNKVTLA	HLA-DPA1*02:01/DPB1*05:01	815	829	Consensus	1.4	Non-antigen (−0.0341)	Allergen	Non-toxic
29	GWTFGAGAALQIPFA	HLA-DRB1*09:01	885	899	Consensus	0.35	Non-antigen (0.4665)	Non-allergen	Non-toxic
30	PREGVFVSNGTHWFV	HLA-DRB1*13:02	1090	1104	Consensus	1.2	Antigen (1.0165)	Non-allergen	Non-toxic
31	REGVFVSNGTHWFVT	HLA-DRB3*02:02	1091	1105	NetMHCIIpan	0.2	Antigen (1.0165)	Non-allergen	Non-toxic
32	SGNCDVVIGIVNNTV	HLA-DRB1*13:02	1123	1137	Consensus	1.3	Antigen (0.5968)	Non-allergen	Non-toxic
33	CDVVIGIVNNTVYDP	HLA-DRB1*13:02	1126	1140	Consensus	0.7	Antigen (0.7320)	Non-allergen	Non-toxic
34	WYIWLGFIAGLIAIV	HLA-DQA1*05:01/DQB1*03:01	1214	1228	Consensus	0.58	Antigen (0.5770)	Non-allergen	Non-toxic
**Indian Variant**									
35	MFVFLVLLPLVSSQC	HLA-DRB1*01:01	1	15	Consensus	0.24	Antigen (0.5741)	Non-allergen	Non-toxic
36	DLFLPFFSNVTWFHA	HLA-DRB1*04:01	53	67	Consensus	1.1	Non-antigen (0.2472)	Non-allergen	Non-toxic
37	KTQSLLIVNNATNVV	HLA-DRB1*13:02	113	127	Consensus	0.01	Antigen (0.6303)	Allergen	Non-toxic
38	REFVFKNIDGYFKIY	HLA-DRB5*01:01	190	204	Consensus	0.17	Non-antigen (−0.1712)	Allergen	Non-toxic
39	TRFASVYAWNRKRIS	HLA-DPA1*02:01/DPB1*14:01	232	246	Consensus	0.52	Non-antigen (0.4963)	Allergen	Non-toxic
40	NYNYLYRLFRKSNLK	HLA-DRB1*11:01	448	462	Consensus	0.42	Non-antigen (0.1089)	Allergen	Non-toxic
41	PYRVVVLSFELLHAP	HLA-DPA1*01:03/DPB1*02:01	507	521	Consensus	0.36	Antigen (0.8161)	Non-allergen	Non-toxic
42	AIPINFTISVTTEIL	HLA-DRB1*07:01	713	727	Consensus	0.29	Antigen (1.1305)	Non-allergen	Non-toxic
43	LQIPFAMQMAYRFNG	HLA-DRB4*01:01	894	908	Consensus	0.73	Antigen (0.7205)	Non-allergen	Non-toxic
44	QQLIRAAEIRASANL	HLA-DPA1*02:01/DPB1*14:01	1010	1024	NetMHCIIpan	0.2	Non-antigen (0.1269)	Allergen	Non-toxic
45	REGVFVSNGTHWFVT	HLA-DRB3*02:02	1091	1195	NetMHCIIpan	0.2	Non-antigen (0.4461)	Allergen	Non-toxic
46	IWLGFIAGLIAIVMV	HLA-DQA1*05:01/DQB1*03:01	1216	1230	Consensus	0.51	Antigen (0.6150)	Non-allergen	Non-toxic
**South African Variant**									
47	MFVFLVLLPLVSSQC	HLA-DRB1*01:01	1	15	Consensus	0.24	Antigen (0.5741)	Non-allergen	Non-toxic
48	FVFLVLLPLVSSQCV	HLA-DRB1*01:01	2	16	Consensus	0.24	Antigen (0.7185)	Non-allergen	Non-toxic
49	LHSTQDLFLPFFSNV	HLA-DPA1*01:03/DPB1*02:01	48	62	Consensus	1.4	Non-antigen (0.2110)	Allergen	Non-toxic
50	KTQSLLIVNNATNVV	HLA-DRB1*13:02	113	127	Consensus	0.01	Antigen (0.6303)	Allergen	Non-toxic
51	REFVFKNIDGYFKIY	HLA-DRB5*01:01	190	204	Consensus	0.17	Non-antigen (−0.1712)	Allergen	Non-toxic
52	NITRFQTLLALHRSY	HLA-DRB5*01:01	234	248	Consensus	0.32	Non-antigen (0.1775)	Non-allergen	Non-toxic
53	ATRFASVYAWNRKRI	HLA-DRB5*01:01	344	358	Consensus	0.49	Non-antigen (0.3489)	Allergen	Non-toxic
54	NYNYLYRLFRKSNLK	HLA-DRB1*11:01	448	462	Consensus	0.42	Non-antigen (0.1089)	Allergen	Non-toxic
55	PYRVVVLSFELLHAP	HLA-DPA1*02:01/DPB1*01:01	507	521	Consensus	0.3	Antigen (0.8161)	Non-allergen	Non-toxic
56	IAIPTNFTISVTTEI	HLA-DRB1*07:01	712	726	Consensus	0.47	Antigen (0.7719)	Non-allergen	Non-toxic
57	TSGWTFGAGAALQIP	HLA-DRB1*09:01	883	897	Consensus	0.34	Non-antigen (−0.0178)	Non-allergen	Non-toxic
58	ALQIPFAMQMAYRFN	HLA-DRB4*01:01	893	907	Consensus	0.81	Antigen (1.0112)	Allergen	Non-toxic
59	QQLIRAAEIRASANL	HLA-DPA1*02:01/DPB1*14:01	1010	1024	NetMHCIIpan	0.2	Non-antigen (0.1269)	Allergen	Non-toxic
60	REGVFVSNGTHWFVT	HLA-DRB3*02:02	1091	1105	NetMHCIIpan	0.2	Non-antigen (0.4461)	Non-allergen	Non-toxic
61	CDVVIGIVNNTVYDP	HLA-DRB1*13:02	1126	1140	Consensus	0.7	Antigen (0.7320)	Non-allergen	Non-toxic
62	YIWLGFIAGLIAIVM	HLA-DQA1*05:01/DQB1*03:01	1215	1229	Consensus	0.51	Antigen (0.6090)	Non-allergen	Non-toxic

**Table 4:** Potential linear B-cell epitopes identified in each variant predicted by Ellipro.

Position	Epitope Sequence	Score	Antigenicity
**Wuhan Isolate**			
14–28	QCVNLTTRTQLPPAY	0.772	1.4548
109–114	TLDSKT	0.529	1.1073
1033–1039	VLGQSKR	0.523	1.6008
**England Isolate**			
392–429	FTNVYADSFVIRGDEVRQIAPGQTGKIADYNYKLPDDF	0.695	0.5786
576–585	VRDPQTLEIL	0.644	0.5446
872–928	QYTSALLAGTITSGWTFGAGAALQIPFAMQMAYRFNGIGVTQNVLYENQKLIANQFN	0.649	0.5394
**USA Isolate**			
392–429	FTNVYADSFVIRGDEVRQIAPGQTGKIADYNYKLPDDF	0.695	0.5786
553–565	TESNKKFLPFQQF	0.666	0.5056
872–928	QYTSALLAGTITSGWTFGAGAALQIPFAMQMAYRFNGIGVTQNVLYENQKLIANQFN	0.649	0.5394
576–585	VRDPQTLEIL	0.644	0.5449
**Indian Isolate**			
239–265	QTLLALHRSYLTPGDSSSGWTAGAAAY	0.816	0.4822
14–27	QCVNLTTRTQLPPA	0.771	1.4983
64–83	WFHAGASSGTNGTKRFDNPV	0.763	0.4097
169–190	EYVSQPFLMDLEGKQGNFKNLR LIVNNATNVVIKVCEFQFCNDPFLGVYYHKNNKSWMESEFRVYSSANNCT	0.75	0.7830
118–167	RFPNITNLCPFGEVFNATRFASVYAWNRKRISNCVADYSVLYNSASFSTFK	0.732	0.3023
**South African Isolate**			
239–265	QTLLALHRSYLTPGDSSSGWTAGAAAY	0.815	0.4822
14–27	QCVNLTTRTQLPPA	0.769	1.4983
64–83	WFHAIHVSGTNGTKRFDNPV	0.763	0.4100
169–190	EYVSQPFLMDLEGKQGNFKNLR	0.75	0.7830
118–167	LIVNNATNVVIKVCEFQFCNDPFLGVYYHKNNKSWMESEFRVYSSANNCT	0.731	0.1177
328–378	RFPNITNLCPFGEVFNATRFASVYAWNRKRISNCVADYSVLYNSASFSTFK	0.728	0.3023

**Table 5:** Discontinuous B-cell epitopes of SARS-CoV variants predicted by IEDB-DiscoTope.

Residue Position	Residue Name	Contact Number	Propensity Score	DiscotopeScore
Wuhan Isolate				
181	GLY	6	0.026	−0.667
183	GLN	19	1.817	−0.577
444	LYS	9	1.701	0.47
447	GLY	5	1.651	0.886
449	TYR	4	−0.223	−0.667
496	GLY	3	0.343	−0.041
501	ASN	27	3.051	−0.405
679	ASN	15	1.01	−0.831
684	ALA	11	1.663	0.206
1144	GLU	7	0.215	−0.615
1145	LEU	4	−0.092	−0.541
**England Isolate**				
72	GLY	11	0.723	−0.625
75	GLY	10	1.381	0.072
147	LYS	10	1.503	0.18
148	ASN	13	1.34	−0.309
149	ASN	17	1.084	−0.996
152	TRP	14	2.444	0.553
498	GLN	6	0.354	−0.377
499	PRO	9	1.027	−0.126
1142	GLN	7	0.467	−0.392
1144	GLU	3	1.177	0.697
1145	LEU	5	0.608	−0.037
1147	SER	6	0.413	−0.325
1148	PHE	5	0.591	−0.052
**USA Isolate**				
72	GLY	11	0.718	−0.629
75	GLY	10	1.379	0.071
147	LYS	10	1.502	0.179
148	ASN	13	1.336	−0.313
150	LYS	10	2.307	0.891
152	TRP	14	2.443	0.552
154	GLU	2	−0.24	−0.442
444	LYS	9	0.883	−0.253
447	GLY	6	0.901	0.107
496	GLY	4	0.289	−0.204
498	GLN	6	1.323	0.481
499	PRO	9	1.995	0.731
501	ASN	24	3.012	−0.095
1141	LEU	3	−0.549	−0.831
1142	GLN	7	0.466	−0.393
1145	LEU	5	0.608	−0.037
1147	SER	6	0.412	−0.325
1148	PHE	5	0.59	−0.053
1149	LYS	5	0.797	0.13
**Indian Isolate**				
147	LYS	10	4.318	−0.682
149	ASN	9	4.399	−0.101
153	MET	18	1.485	−7.515
424	LYS	24	4.315	−7.685
460	ASN	18	2.804	−6.196
461	LEU	17	3.048	−5.452
462	LYS	16	3.219	−4.781
501	TYR	15	1.556	−5.944
563	GLN	11	1.367	−4.133
679	ASN	10	3.455	−1.545
809	PRO	11	4.234	−1.266
1158	ASN	10	4.027	−0.973
1159	HIS	10	4.027	−0.973
1160	THR	8	3.607	−0.393
1161	SER	7	3.308	−0.192
**South African Isolate**				
146	HIS	14	3.276	3.724
147	LYS	11	4.084	−1.416
148	ASN	8	4.194	0.194
149	ASN	9	4.399	−0.101
150	LYS	8	4.194	0.194
151	SER	13	4.108	−2.392
152	TRP	18	3.747	−5.253
409	GLN	20	2.989	−7.011
414	GLN	18	2.979	−6.021
424	LYS	9	4.399	−0.101
498	GLN	21	3.461	−7.039
499	PRO	20	3.536	−6.464
501	ASN	15	2.768	−4.732
679	ASN	9	3.115	−1.385
680	SER	9	3.115	−1.385
809	PRO	11	4.234	−1.266
810	SER	13	3.958	−0.542
811	LYS	12	3.646	−2.354
1155	TYR	11	2.84	−2.66
1156	PHE	12	3.55	−2.45
1157	LYS	10	3.353	−1.647
1158	ASN	10	4.027	−0.973
1159	HIS	10	4.027	−0.973
1160	THR	8	3.607	−0.393
1160	THR	8	3.607	−0.393
1161	SER	7	3.308	−0.192

**Table 6:** Population coverage and the distribution of immunogenic T-cell epitopes of SARS-CoV-2 variants.

Epitope	Country	Population Coverage
Wuhan Strain		
ILDITPCSF STQDLFLPF VVFLHVTYV GVVFLHVTY WTAGAAAYY GAAAYYVGY	World	93.65%
	South Asia	88.23%
	India	80.22%
	England	97.08%
	France	96.42%
	Italy	95.12%
	Sweden	92.66%
	United States	95.3
	South Africa	87.07%
**England Isolate**		
NGVEGFNCY QYIKWPWYI WTAGAAAYY VYAWNRKRI GVYFASTEK SPRRARSVA PYRVVVLSF	World	94.15%
	South Asia	92.78%
	India	89.3%
	England	97.62%
	France	97.36%
	Italy	95.84%
	Sweden	96.12%
	United States	93.76%
	South Africa	96.49%
**USA Isolate**		
VVFLHVTYV IAIVMVTIM LPFNDGVYF IAIPTNFTI	World	91.98%
	South Asia	81.6%
	India	73.8%
	England	95.97%
	France	96.2%
	Italy	93.59%
	Sweden	98.77%
	United States	94.9%
	South Africa	83.62%
**Indian Isolate**		
FTISVTTEI VVFLHVTYV YQPYRVVVL YSKHTPINL WTAGAAAYY LPFNDGVYF GAAAYYVGY	World	97.98%
	South Asia	91.95%
	India	85.34%
	England	99.71%
	France	99.21%
	Italy	97.97%
	Sweden	99.82%
	United States	98.07%
	South Africa	90.51%
**South African Isolate**		
IAIPINFTI FTISVTTEI YQPYRVVVL WTAGAAAYY YSKHTPINL GVVFLHVTY LPFNDGVYF	World	97.48%
	South Asia	91.95%
	India	85.34%
	England	99.71%
	France	99.21%
	Italy	97.97%
	Sweden	99.82%
	United States	98.07%
	South Africa	90.51%

**Table 7:** Binding interactions of the best T-cell epitopes of the SARS-CoV-2 variants with their MHC I alleles.

S.No.	Potential Peptide for Vaccine	Binding Alleles	Attractive vdW	Repulsive vdW	ACE	HB	Global Energy
Wuhan Isolate							
1.	ILDITPCSF	HLA-B*51:01	−28.64	7.59	−9.54	−2.23	−58.28
		HLA-B*08:01	−24.90	5.90	−8.27	−3.92	−50.66
		HLA-A*02:06	−24.14	10.32	−9.30	−2.48	−49.01
2.	STQDLFLPF	HLA-A*32:01	−19.73	14.97	−11.66	−1.83	−44.18
		HLA-B*57:01	−29.53	21.42	−3.31	−3.03	−40.05
		HLA-B*15:01	−3.50	0.00	0.36	0.00	−8.53
3.	VVFLHVTYV	HLA-B*51:01	−29.19	10.45	−1.43	−3.35	−51.05
		HLA-A*02:03	−18.26	2.58	−9.10	−4.13	−42.85
4.	GVVFLHVTY	HLA-B*35:01	−36.19	7.98	−8.72	−1.99	−66.28
		HLA-A*01:01	−32.13	6.50	−7.59	−2.76	−55.83
		HLA-B*15:01	−9.36	2.62	0.96	−0.20	−3.98
5.	WTAGAAAYY	HLA-B*35:01	−27.77	4.34	−2.83	−3.28	−54.98
		HLA-A*01:01	−30.65	13.59	−7.64	−4.80	−53.52
6.	GAAAYYVGY	HLA-A*30:02	−25.83	3.78	−4.26	−4.52	−51.90
		HLA-B*15:01	−20.22	7.80	−0.58	−0.99	−24.32
**England Isolate**							
7.	QYIKWPWYI	HLA-A*23:01	−25.46	8.91	−13.16	−0.95	−57.28
		HLA-C*06:02	−29.14	16.66	−4.45	−3.18	−50.48
8.	GVYFASTEK	HLA-A*30:01	−21.73	4.79	2.52	−3.41	−28.36
9.	NGVEGFNCY	HLA-B*35:01	−37.69	6.42	2.02	−2.25	−46.33
10.	PYRVVVLSF	HLA-A*23:01	−25.49	9.71	−11.33	−2.48	−65.01
		HLA-C*14:02	−25.48	5.04	−1.94	−0.95	−41.16
11.	VYAWNRKRI	HLA-A*23:01	−22.44	4.18	−4.02	−3.34	−46.75
12.	SPRRARSVA	HLA-B*07:02	−25.14	8.95	3.94	−1.97	−18.83
**USA Isolate**							
13.	IAIVMVTIM	HLA-B*51:01	−30.57	25.38	−16.59	−0.98	−59.86
14.	LPFNDGVYF	HLA-B*35:01	−35.26	47.18	−3.06	−3.78	−30.68
**Indian Isolate**							
15.	YQPYRVVVL	HLA-B*08:01	−36.00	10.43	−7.97	−2.46	−62.85
16.	YSKHTPINL	HLA-A*68:02	−22.93	6.44	−10.43	−2.56	−59.11
17.	FTISVTTEI	HLA-A*68:02	−29.71	11.47	−7.29	−2.07	−53.01
18.	WTAGAAAYY	HLA-A*26:01	−31.19	8.71	−0.08	−1.66	−44.75
19.	GAAAYYVGY	HLA-A*30:02	−19.74	5.30	−9.59	−2.21	−43.65
**South African Isolate**							
20.	IAIPINFTI	HLA-B*51:01	−22.76	6.82	−13.73	−0.80	−54.96
21.	FTISVTTEI	HLA-B*51:01	−22.68	11.47	−7.29	−7.29	−53.01
22.	QLTPTWRVY	HLA-B*35:01	−19.09	8.37	−10.39	0.00	−44.39
23.	YSKHTPINL	HLA-B*57:01	−24.90	3.82	−1.65	−1.65	−41.72
24.	YQPYRVVVL	HLA-B*08:01	−21.47	10.07	−9.41	−0.98	−38.80

## Data Availability

Data available upon request by the corresponding author.

## Supplementary Materials

The following are available online at https://www.mdpi.com/article/10.3390/antibiotics10050535/s1, Figure S1: Variability Analysis Plot, Figures S2–S6: Population Coverage Analysis, Figure S7: Protein-Peptide docking interaction site analysis, Figure S8: Protein-Peptide docking interaction site analysis, Table S1: Conserved/Variabile regions in sequences, Table S2: Potential CTL epitopes identified in each variant studied and Table S3: Predicted B-cell linear epitopes.
