# Supplementary material for: Comparison of Immunological Profiles of SARS-CoV-2 Variants in the COVID-19 Pandemic Trends: An Immunoinformatics Approach
Source: Antibiotics (Basel). 2021 May 6;10(5):535. doi: 10.3390/antibiotics10050535 (PMC8148159; doi:10.3390/antibiotics10050535)
Supplement: Supplementary file 1 [file antibiotics-10-00535-s001.zip › Supplementary Figure S1.pdf]

Supplementary Figure S1 Variability analysis plot

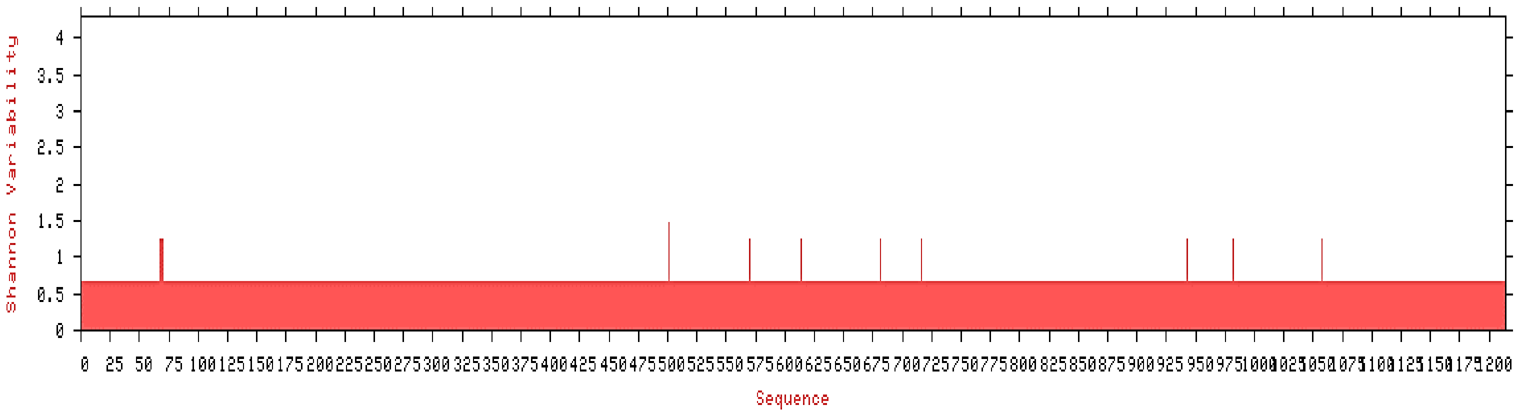

Figure S1. Variability Analysis histogram indicates variability less than 1.5 with 6 or more consecutive residues with variability less than 1 and 10 large fragments of the sequence to beconserved among the five SARS-CoV-2 isolates studied. X-axis represents ‘sequence’ and Y-axis represents ‘sequence variability’. Variability threshold was set as 1 and base sequence bar denotes consensus.
