# Supplementary material for: Comparison of Immunological Profiles of SARS-CoV-2 Variants in the COVID-19 Pandemic Trends: An Immunoinformatics Approach
Source: Antibiotics (Basel). 2021 May 6;10(5):535. doi: 10.3390/antibiotics10050535 (PMC8148159; doi:10.3390/antibiotics10050535)
Supplement: Supplementary file 1 [file antibiotics-10-00535-s001.zip › Supplementary Figure S2-S6.pdf]

Supplementary Figure S2 Population coverage analysis

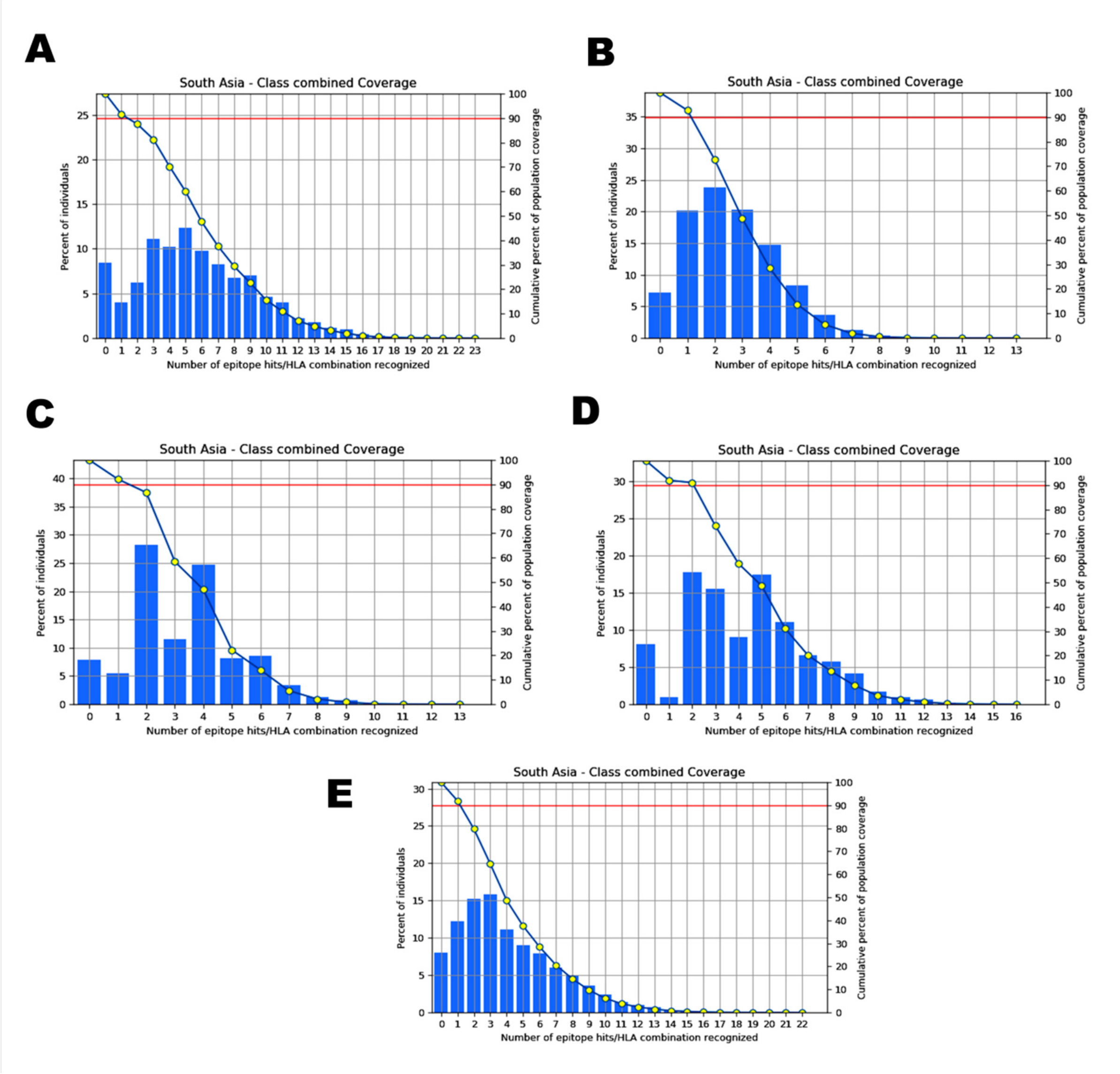

Figure S2 Graphs depicting the coverage of MHC I alleles for the South Asian population of the spike glycoprotein of SARS-CoV-2 variants isolated from A) Wuhan, China, B) England, C) USA, D) India, and E) South Africa respectively.

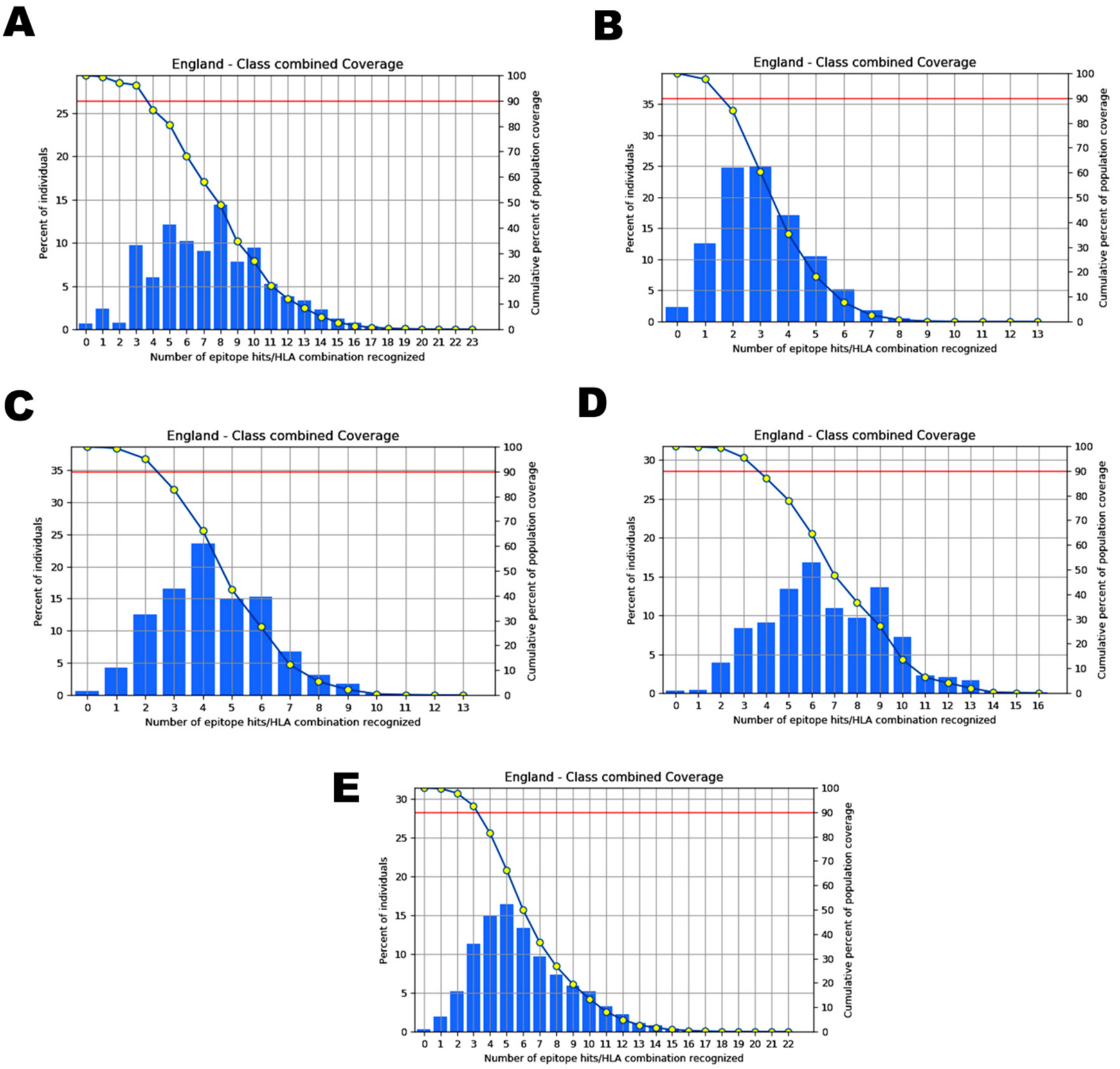

Figure S3 Graphs depicting the coverage of MHC I alleles for the England population of the spike glycoprotein of SARS-CoV-2 variants isolated from A) Wuhan, China, B) England, C) USA, D) India, and E) South Africa respectively.

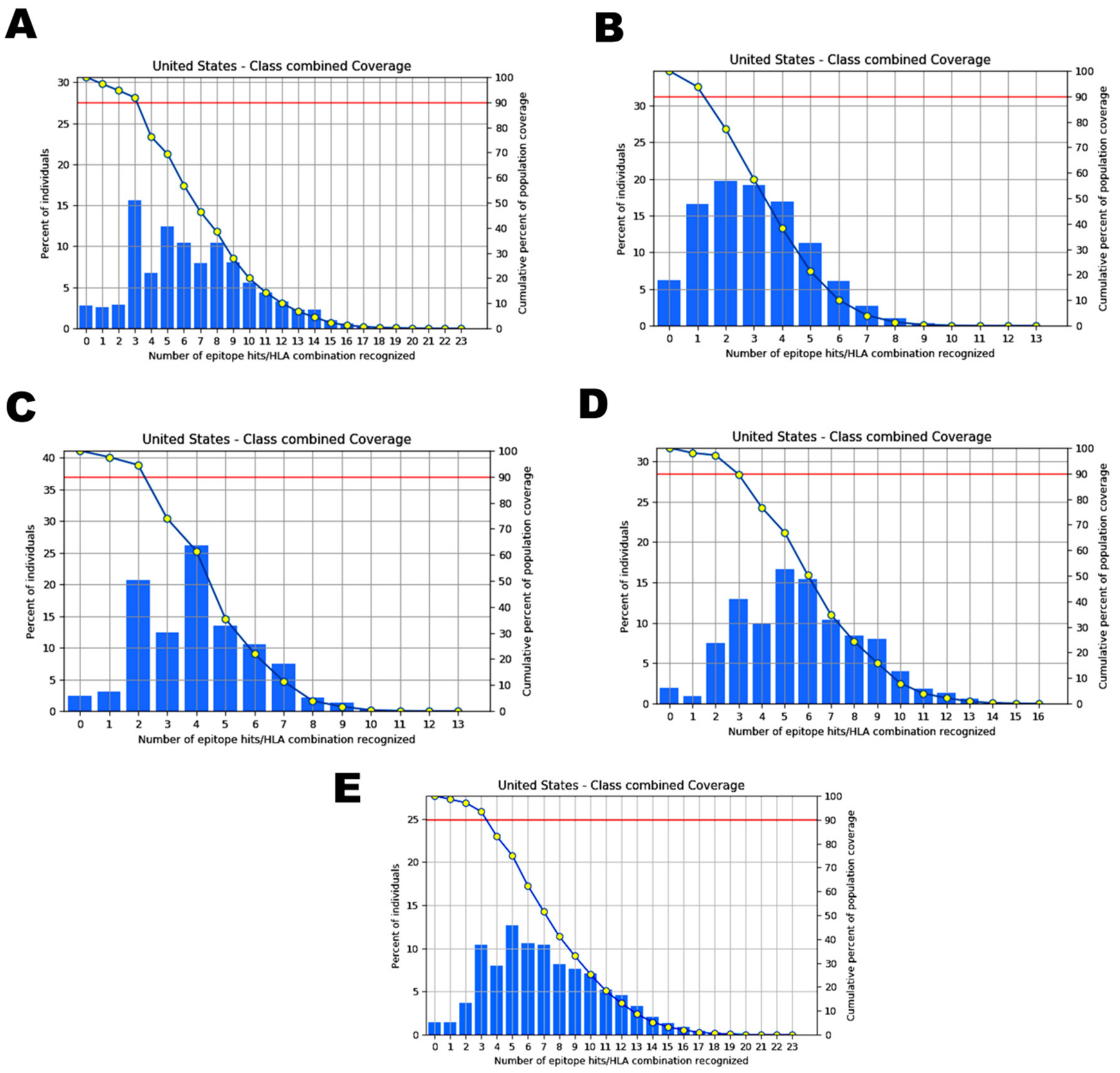

Figure S4 Graphs depicting the coverage of MHC I alleles for the USA population of the spike glycoprotein of SARS-CoV-2 variants isolated from A) Wuhan, China, B) England, C) USA, D) India and E) South Africa respectively.

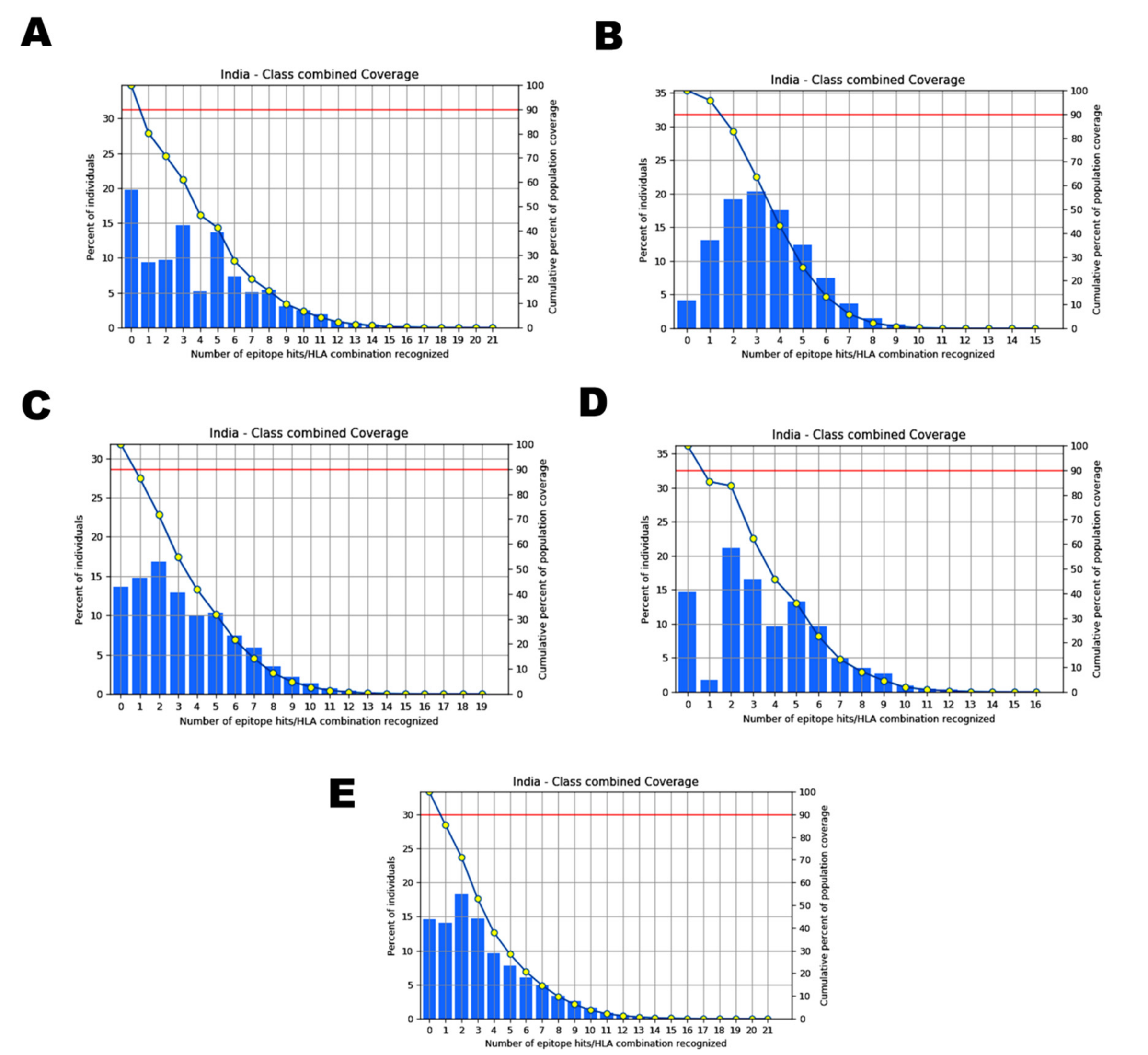

Figure S5 Graphs depicting the coverage of MHC I alleles for the Indian population of the spike glycoprotein of SARS-CoV-2 variants isolated from A) Wuhan, China, B) England, C) USA, D) India and E) South Africa respectively.

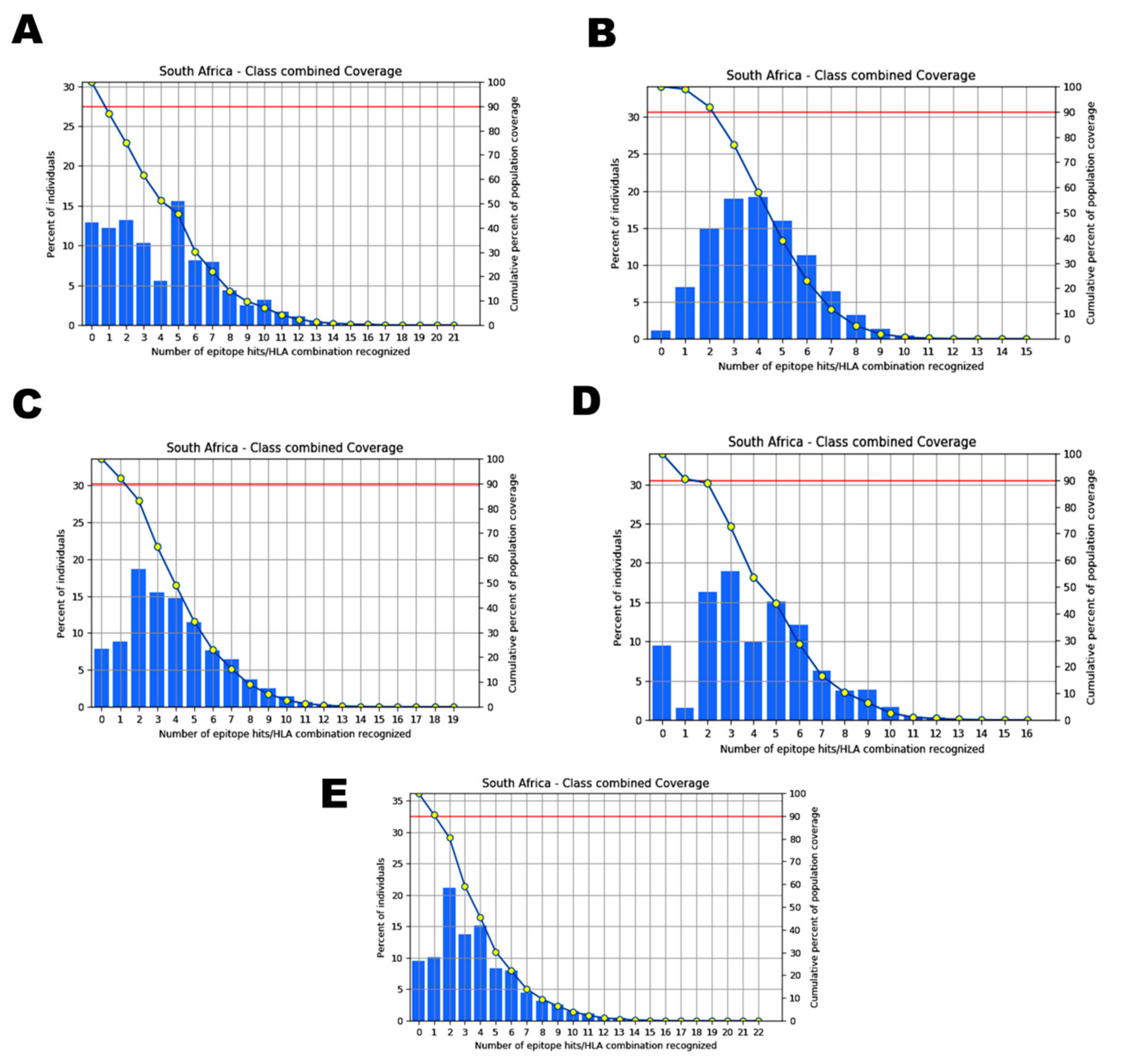

Figure S6 Graphs depicting the coverage of MHC I alleles for the South African population of the spike glycoprotein of SARS-CoV-2 variants isolated from A) Wuhan, China, B) England, C) USA, D) India and E) South Africa respectively.
