# Supplementary material for: Comparison of Immunological Profiles of SARS-CoV-2 Variants in the COVID-19 Pandemic Trends: An Immunoinformatics Approach
Source: Antibiotics (Basel). 2021 May 6;10(5):535. doi: 10.3390/antibiotics10050535 (PMC8148159; doi:10.3390/antibiotics10050535)
Supplement: Supplementary file 1 [file antibiotics-10-00535-s001.zip › Supplementary Figure S7-S8.pdf]

Supplementary Figures S7 Protein-peptide docking interaction complexes of the best epitopes identified in Wuhan, England, and the USA

Hydrogen bond interactions and the interacting amino acids of each complex in the binding pocket of the HLA proteins are highlighted in the illustrations given below

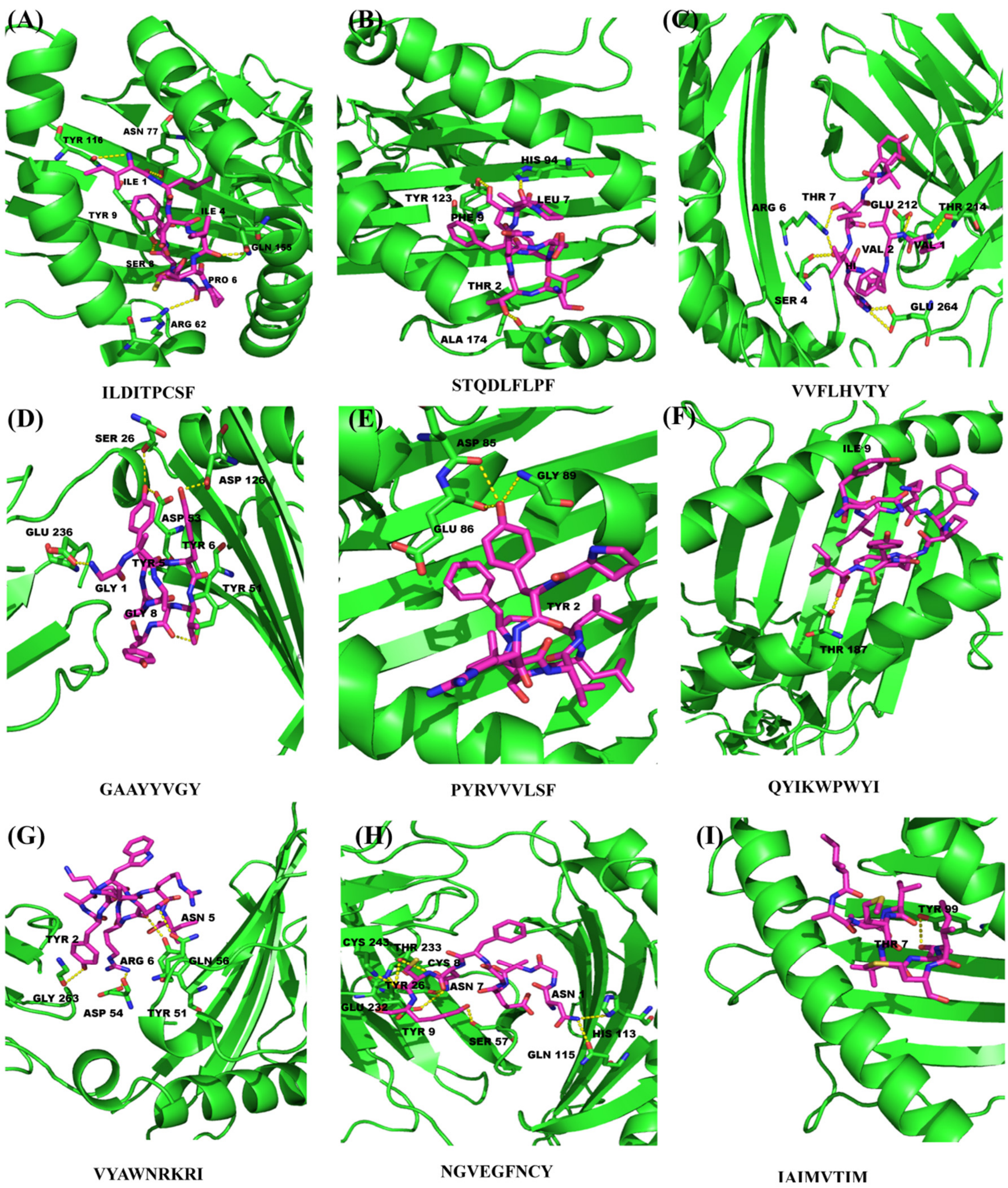

Figure S7 Protein-peptide interactions A-C) Epitope-Allele complexes of Wuhan strain and D-F) Epitope-Allele complexes of England variant, and G-I) Epitope-Allele complexes of the USA isolate respectively.

Supplementary Figures S8 Protein-peptide docking interaction complexes of the best epitopes identified in India and South Africa

Hydrogen bond interactions and the interacting amino acids of each complex in the binding pocket of the HLA proteins are highlighted in the illustrations given below

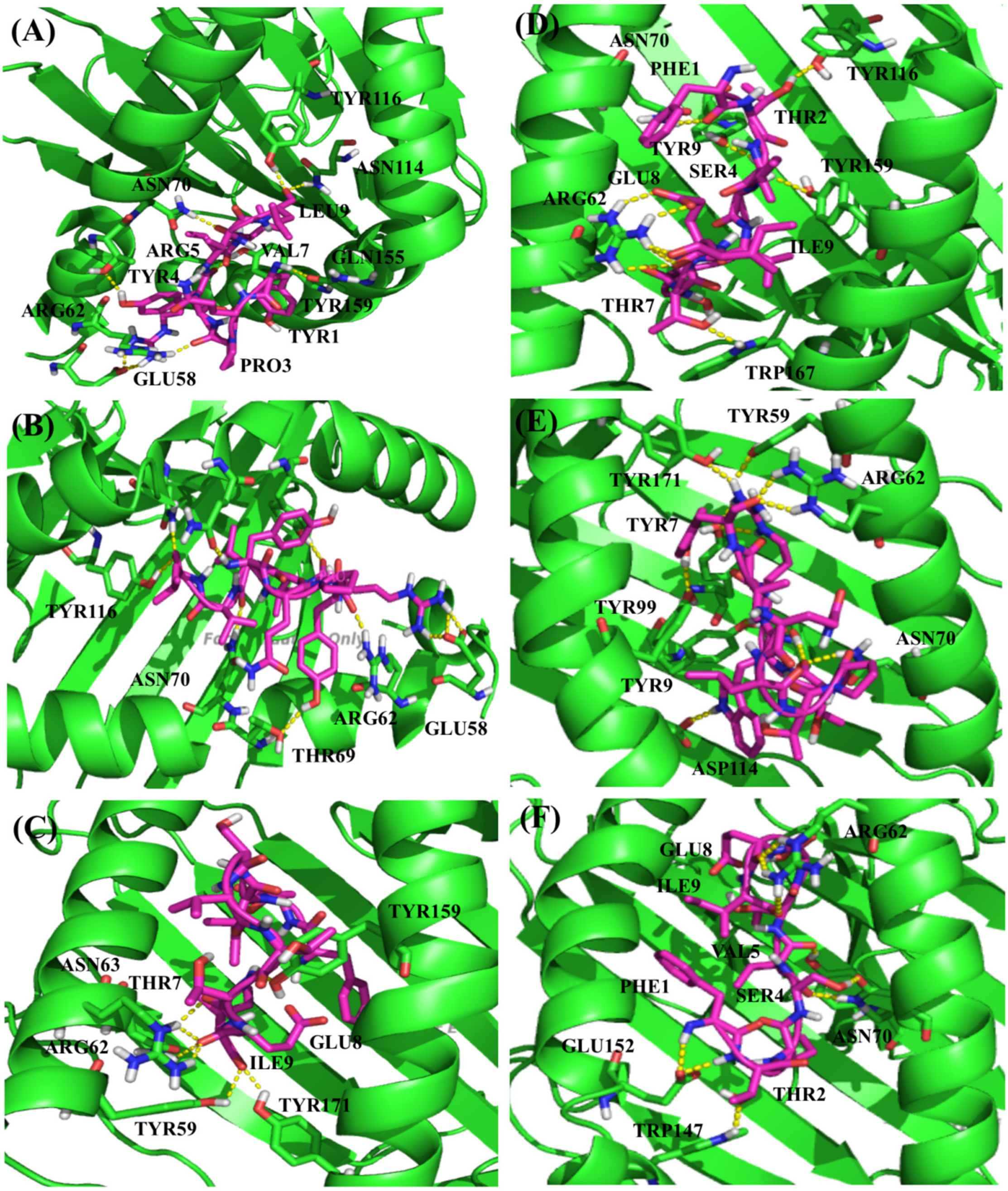

Figure S8 Protein-peptide interactions A-C) Epitope-Allele complexes of Indian variant and D-F) Epitope-Allele complexes of South African variant respectively.
