# Supplementary material for: Comparison of Immunological Profiles of SARS-CoV-2 Variants in the COVID-19 Pandemic Trends: An Immunoinformatics Approach
Source: Antibiotics (Basel). 2021 May 6;10(5):535. doi: 10.3390/antibiotics10050535 (PMC8148159; doi:10.3390/antibiotics10050535)
Supplement: Supplementary file 1 [file antibiotics-10-00535-s001.zip › Supplementary Table S1.pdf]

Supplementary Table S1 Conserved regions and variability of SARS-CoV-2 genomes isolated from different geographical regions of the world. Consensus of highly conserved sequence fragments with variability factor less than 1 are presented in this table below.

Fragments of 6 or more consecutive residues with V <= 1

N	Start	End	Sequence
1	1	67	M F V F L V L L P L V S S Q C V N L T T R T Q L P P A Y T N S F T R<br>G V Y Y P D K V F R S S V L H S T Q D L F L P F F S N V T W F H A
2	71	500	S G T N G T K R F D N P V L P F N D G V Y F A S T E K S N I I R G W<br>I F G T T L D S K T Q S L L I V N N A T N V V I K V C E F Q F C N D P<br>F L G V Y Y H K N N K S W M E S E F R V Y S S A N N C T F E Y V S<br>Q P F L M D L E G K Q G N F K N L R E F V F K N I D G Y F K I Y S K<br>H T P I N L V R D L P Q G F S A L E P L V D L P I G I N I T R F Q T L L<br>A L H R S Y L T P G D S S S G W T A G A A A Y Y V G Y L Q P R T F<br>L L K Y N E N G T I T D A V D C A L D P L S E T K C T L K S F T V E<br>K G I Y Q T S N F R V Q P T E S I V R F P N I T N L C P F G E V F N A<br>T R F A S V Y A W N R K R I S N C V A D Y S V L Y N S A S F S T F K<br>C Y G V S P T K L N D L C F T N V Y A D S F V I R G D E V R Q I A P<br>G Q T G K I A D Y N Y K L P D D F T G C V I A W N S N N L D S K V<br>G G N Y N Y L Y R L F R K S N L K P F E R D I S T E I Y Q A G S T P<br>C N G V E G F N C Y F P L Q S Y G F Q P T
3	502	569	G V G Y Q P Y R V V V L S F E L L H A P A T V C G P K K S T N L V<br>K N K C V N F N F N G L T G T G V L T E S N K K F L P F Q Q F G R D<br>I
4	571	613	D T T D A V R D P Q T L E I L D I T P C S F G G V S V I T P G T N T S<br>N Q V A V L Y Q
5	615	680	V N C T E V P V A I H A D Q L T P T W R V Y S T G S N V F Q T R A<br>G C L I G A E H V N N S Y E C D I P I G A G I C A S Y Q T Q T N S
6	682	715	R R A R S V A S Q S I I A Y T M S L G A E N S V A Y S N N S I A I P
7	717	942	N F T I S V T T E I L P V S M T K T S V D C T M Y I C G D S T E C S N<br>L L L Q Y G S F C T Q L N R A L T G I A V E Q D K N T Q E V F A Q V<br>K Q I Y K T P P I K D F G G F N F S Q I L P D P S K P S K R S F I E D L<br>L F N K V T L A D A G F I K Q Y G D C L G D I A A R D L I C A Q K F<br>N G L T V L P P L L T D E M I A Q Y T S A L L A G T I T S G W T F G<br>A G A A L Q I P F A M Q M A Y R F N G I G V T Q N V L Y E N Q K L<br>I A N Q F N S A I G K I Q D S L S S T A
8	944	981	A L G K L Q D V V N Q N A Q A L N T L V K Q L S S N F G A I S S V<br>L N D I L
9	983	1057	R L D K V E A E V Q I D R L I T G R L Q S L Q T Y V T Q Q L I R A A<br>E I R A I C H D G K A H F P R E G V F V S N G T H W F V T Q R N F Y<br>E P Q I I T T
10	1058	1212	N T F V S G N C D V V I G I V N N T V Y D P L Q P E L D S F K E E L

			D K Y F K N H T S P D V D L G D I S G I N A S V V N I Q K E I D R L N E V A K N L N E S L I D L Q E L G K Y E Q Y I K W P W Y I W L G F I A G L I A I V M V T I M L C C M T S C C S C L K G C C S C G S C C K F D E D D S E P V L K G V K L H Y T
