# Supplementary material for: Comparison of Immunological Profiles of SARS-CoV-2 Variants in the COVID-19 Pandemic Trends: An Immunoinformatics Approach
Source: Antibiotics (Basel). 2021 May 6;10(5):535. doi: 10.3390/antibiotics10050535 (PMC8148159; doi:10.3390/antibiotics10050535)
Supplement: Supplementary file 1 [file antibiotics-10-00535-s001.zip › Supplementary Table S2.pdf]

Supplementary Table S2 List of CTL epitope 9mers and their immunogenic characteristics predicted from the spike glycoprotein of the three different SARS-CoV-2 isolates. Antigenic, immunogenic, non-allergic/allergic, and non-toxic/toxic CTL epitopes and their HLA-combinations identified in the S protein of each variant are presented in this table.

SARS-CoV-2 Variant	Epitope	Position	Antigenicity score	Immunogenicity Score	MHC I alleles	No of MHC I binding alleles	Conservancy at 100% sequence identity	Allergenicity	Toxicity
Wuhan, China	ILDITPCSF	584-592	1.184	0.02632	HLA-B*15:01, HLA-A*01:01, HLA-A*02:06, HLA-B*35:01, HLA-B*08:01, HLA-A*02:01, HLA-A*32:01, HLA-A*24:02, HLA-A*23:01, HLA-A*30:02, HLA-B*58:01, HLA-B*53:01	12	100%	Non-allergen	Non-toxic
	STQDLFLPF	50-58	0.662	0.06828	HLA-A*32:01, HLA-B*57:01, HLA-B*15:01, HLA-A*26:01, HLA-B*58:01, HLA-B*35:01, HLA-A*30:02, HLA-A*23:01, HLA-A*24:02, HLA-A*01:01, HLA-A*11:01, HLA-B*53:01	12	100%	Non-allergen	Non-toxic
	VVFLHVTYV	1060-1068	1.512	0.1278	HLA-A*02:06, HLA-A*02:03, HLA-A*02:01, HLA-A*68:02, HLA-B*51:01, HLA-A*30:01, HLA-A*32:01, HLA-B*08:01, HLA-A*26:01	9	100%	Non-allergen	Non-toxic
	GVVFLHVTY	1059-1067	1.410	0.20837	HLA-B*15:01, HLA-A*30:02, HLA-A*26:01, HLA-B*35:01,	9	100%	Non-allergen	Non-toxic

					HLA-A*32:01, HLA-B*57:01, HLA-A*11:01, HLA-A*01:01, HLA-B*58:01				
	WTAGAAAYY	258-266	0.662	0.152 59	HLA-A*26:01, HLA-A*01:01, HLA-A*30:02, HLA-A*68:01, HLA-B*35:01, HLA-B*15:01, HLA-B*58:01, HLA-B*57:01	8	100%	Non-allergen	Non-toxic
	GAAAYYVGY	261-269	0.660	0.099 63	HLA-A*30:02, HLA-B*15:01, HLA-B*35:01, HLA-A*26:01, HLA-A*01:01, HLA-A*11:01, HLA-B*58:01	7	100%	Non-allergen	Non-toxic
	TLLALHRSY	240-248	0.801	0.002 44	HLA-B*15:01, HLA-A*30:02, HLA-A*32:01, HLA-B*35:01, HLA-B*57:01, HLA-A*03:01, HLA-A*26:01	7	100%	Allergen	Non-toxic
	KEIDRLNEV	1181-1189	0.530	0.158 52	HLA-B*40:01, HLA-B*44:03, HLA-B*44:02, HLA-A*02:06, HLA-A*02:03, HLA-A*02:01	6	100%	Allergen	Non-toxic
	RVVVLSFEL	509-517	1.192	0.046	HLA-A*32:01, HLA-A*02:06, HLA-B*57:01, HLA-B*58:01, HLA-A*02:01	5	100%	Non-allergen	Non-toxic
	AEIRASANL	1016-1024	0.708	0.006 89	HLA-B*40:01, HLA-B*44:03, HLA-B*44:02	3	100%	Allergen	Non-toxic
	TRTQLPPAY	20-28	1.292	0.224 364	HLA-A*30:02, HLA-B*35:01	2	100%	Allergen	Non-toxic
	DIADTTDAV	568-576	1.090	0.150 94	HLA-A*68:02, HLA-A*26:01	2	100%	Allergen	Non-toxic
England	IAIPTNFTI	712-720	0.523	0.185 23	HLA-B*51:01, HLA-C*17:01, HLA-C*03:04, HLA-C*03:03, HLA-B*58:01, HLA-C*15:02, HLA-B*57:01, HLA-C*03:02, HLA-C*12:03, HLA-C*16:01	10	100%	Allergen	Non-toxic

	WTAGAAAY	258-266	0.826	0.152 59	HLA-A*26:01, HLA-A*01:01, HLA-A*30:02, HLA-A*68:01, HLA-B*35:01, HLA-B*15:01, HLA-B*58:01, HLA-B*53:01, , HLA-B*57:01	9	100%	Non-allergen	Non-toxic
	QLTPTWRVY	628-636	0.935	0.315 55	HLA-B*15:01, HLA-A*30:02, HLA-C*03:02, HLA-A*32:01, HLA-A*26:01, HLA-B*35:01, HLA-C*12:02, HLA-C*02:09, HLA-C*02:02, HLA-A*01:01, HLA-C*16:01, HLA-C*14:02, HLA-C*12:03	8	100%	Allergen	Non-toxic
	QYIKWPWYI	1208-1216	1.664	0.216 24	HLA-A*24:02, HLA-A*23:01, HLA-C*06:02, HLA-C*07:02, HLA-C*14:02, HLA-A*32:01, HLA-C*07:01	7	100%	Non-allergen	Non-toxic
	YQPYRVVVL	505-513	1.421	0.140 9	HLA-C*01:02, HLA-B*08:01, HLA-C*06:02, HLA-C*17:01, HLA-A*02:06	5	100%	Allergen	Non-toxic
	GVYFASTEK	89-97	0.664	0.090 23	HLA-A*11:01, HLA-A*30:01, HLA-A*68:01, HLA-A*31:01	4	100%	Non-allergen	Non-toxic
	NGVEGFNCY	481-489	1.182	0.220 39	HLA-B*35:01, HLA-A*26:01, HLA-C*12:02	3	100%	Non-allergen	Non-toxic
	PYRVVVLSF	507-515	1.028	0.031 38	HLA-A*23:01, HLA-A*24:02, HLA-C*14:02	3	100%	Non-allergen	Non-toxic
	VYAWNRKRI	350-358	0.813	0.126 25	HLA-A*24:02, HLA-C*14:02, HLA-A*23:01	3	100%	Non-allergen	Non-toxic
	SPRRARSVA	680-688	0.511	0.040 2	HLA-B*07:02, HLA-B*08:01	2	100%	Non-allergen	Non-toxic
USA	VVFLHVTYV	1060-1068	1.51	0.127 8	HLA-A*02:06, HLA-A*02:03, HLA-A*02:01, HLA-A*68:02, HLA-B*51:01, HLA-A*30:01,	16	100%	Non-allergen	Non-toxic

					HLA-A*30:02, HLA-A*32:01, HLA-B*08:01, HLA-A*26:01, HLA-A*33:01, HLA-A*03:01, HLA-A*31:01 HLA-B*57:01, HLA-B*15:01, HLA-A*68:01				
	ILDITPCSF	584-592	1.184	0.026 32	HLA-B*15:01, HLA-A*01:01, HLA-A*02:06, HLA-B*35:01, HLA-B*08:01, HLA-A*02:01, HLA-A*32:01, HLA-A*24:02, HLA-A*23:01, HLA-A*30:02, HLA-B*58:01, HLA-B*53:01	12	100%	Non-allergen	Non-toxic
	FTISVTTEI	718-726	0.856	0.044 73	HLA-A*68:02, HLA-A*02:06, HLA-A*02:03, HLA-A*02:01, HLA-B*51:01, HLA-A*26:01, HLA-B*58:01, HLA-B*57:01, HLA-A*32:01, HLA-B*53:01, HLA-B*35:01	11	100%	Allergen	Non-toxic
	IAIPTNFTI	712-720	0.705	0.185 23	HLA-B*51:01, HLA-B*58:01, HLA-B*57:01, HLA-A*02:06, HLA-B*53:01, HLA-A*68:02, HLA-A*32:01, HLA-B*35:01, HLA-A*02:01, HLA-A*23:01, HLA-A*24:02	11	100%	Allergen	Non-toxic
	QLTPTWRVY	628-636	1.212	0.315 55	HLA-B*15:01, HLA-A*30:02, HLA-A*32:01, HLA-B*57:01, HLA-A*26:01, HLA-B*35:01, HLA-A*01:01, HLA-A*03:01, HLA-B*58:01	9	100%	Allergen	Non-toxic
	GVVFLHVTY	1059-1067	1.140	0.208 37	HLA-B*15:01, HLA-A*30:02, HLA-A*26:01,	8	100%	Non-allergen	Non-toxic

					HLA-B*35:01, HLA-A*32:01, HLA-B*57:01, HLA-A*11:01, HLA-B*58:01				
	TLLALHRSY	240-248	0.801	0.00244	HLA-B*15:01, HLA-A*30:02, HLA-A*32:01, HLA-B*35:01, HLA-B*57:01, HLA-A*03:01, HLA-A*26:01, HLA-B*58:01	8	100%	Allergen	Non-toxic
	YQPYRVVVL	505-513	0.596	0.1409	HLA-B*08:01, HLA-A*02:06, HLA-B*15:01, HLA-A*02:03, HLA-A*02:01, HLA-A*24:02, HLA-B*40:01, HLA-A*23:01	8	100%	Allergen	Non-toxic
	GAAAYYVGY	1060-1068	0.661	0.09963	HLA-A*30:02, HLA-B*15:01, HLA-B*35:01, HLA-A*26:01, HLA-A*01:01, HLA-A*11:01, HLA-B*58:01	7	100%	Non-allergen	Non-toxic
	WTAGAAAYY	258-266	0.631	0.15259	HLA-A*26:01, HLA-A*01:01, HLA-A*30:02, HLA-A*68:01, HLA-B*35:01, HLA-B*15:01, HLA-B*58:01	7	100%	Non-allergen	Non-toxic
	LPFNDGVYF	84-92	0.559	0.11767	HLA-B*35:01, HLA-B*53:01, HLA-B*51:01, HLA-B*07:02, HLA-A*26:01	5	100%	Non-allergen	Non-toxic
	IAIVMVTIM	1225-1233	1.134	0.06312	HLA-B*51:01, HLA-B*35:01	2	100%	Non-allergen	Non-toxic
India	FTISVTTEI	718-726	0.8535	0.04473	HLA-A*68:02; HLA-A*02:06; HLA-A*02:03; HLA-A*02:01; HLA-B*51:01; HLA-A*26:01; HLA-B*58:01; HLA-A*32:01; HLA-B*53:01	9	100%	Non – allergen	Non-toxic
	VVFLHVTYV	1060-1068	1.512	0.1278	HLA-A*02:06; HLA-A*02:03; HLA-A*02:01; HLA-A*68:02; HLA-B*51:01;	9	100%	Non-allergen	Non-toxic

					HLA-A*30:01; HLA-A*32:01; HLA-B*08:01; HLA-A*26:01				
	YQPYRVVVL	505-513	0.5964	0.1409	HLA-B*08:01; HLA-A*02:06; HLA-B*15:01; HLA-A*02:03; HLA-A*02:01; HLA-A*24:02; HLA-B*40:01;HLA-A*23:01	8	100%	Non-allergen	Non-toxic
	YSKHTPINL	204-212	1.0547	0.9845	HLA-B*57:01; HLA-A*30:01; HLA-B*08:01; HLA-B*58:01; HLA-A*68:02; HLA-B*51:01; HLA-B*15:01; HLA-A*32:01	8	100%	Non-allergen	Non-toxic
	WTAGAAAYY	258-266	0.6306	0.1525	HLA-A*26:01; HLA-A*01:01; HLA-A*30:02; HLA-A*68:01; HLA-B*35:01; HLA-B*15:01; HLA-B*58:01	7	100%	Non-allergen	Non-toxic
	LPFNDGVYF	84-92	0.5593	0.11767	HLA-B*35:01; HLA-B*53:01; HLA-B*51:01; HLA-B*07:02; HLA-A*26:01	5	100%	Non-allergen	Non-toxic
	GAAAYYVGY	261-269	0.6604	0.9963	HLA-A*30:02; HLA-B*15:01; HLA-B*35:01; HLA-A*26:01; HLA-A*01:01	5	100%	Non-allergen	Non-toxic
	STQDLFLPF	50-58	0.6619	0.06828	HLA-A*32:01 HLA-B*57:01 HLA-B*15:01 HLA-A*26:01 HLA-B*58:01 HLA-B*35:01 HLA-A*30:02 HLA-A*23:01 HLA-A*24:02 HLA-A*01:01 HLA-B*53:01	11	100%	Allergen	Non-toxic
	GVYFASTEK	9-97	0.7112	0.09023	HLA-A*11:01; HLA-A*03:01; HLA-A*68:01; HLA-A*30:01	4	100%	Non-allergen	Non-toxic
	VRFPNITNL	327-355	1.1141	0.1748	HLA-A*23:01; HLA-B*08:01; HLA-A*30:02; HLA-A*24:02	4	100%	Non-allergen	Non-toxic

	TSNQVAVLY	504-612	0.4387	-0.01327	HLA-A*01:01<br>HLA-A*30:02<br>HLA-B*58:01<br>HLA-B*57:01<br>HLA-B*35:01<br>HLA-B*15:01<br>HLA-A*26:01<br>HLA-A*68:01<br>HLA-A*11:01<br>HLA-B*53:01<br>HLA-A*32:01	11	100%	Allergen	Non-toxic
	TLADAGFIK	827-835	0.5781	0.28158	HLA-A*11:01;<br>HLA-A*03:01;<br>HLA-A*68:01	3	100%	Non-allergen	Non-toxic
	AEIRASANL	1016-1024	0.7082	0.00689	HLA-B*40:01;HLA-B*44:03;HLA-B*44:02	3	100%	Non-allergen	Non-toxic
	GQTGKIADY	413-321	1.4019	0.00796	HLA-B*15:01;<br>HLA-A*30:02;<br>HLA-A*26:01	3	100%	Non-allergen	Non-toxic
	SIIAYTMSL	691-699	0.5234	-0.12935	HLA-A*02:06<br>HLA-A*02:01<br>HLA-A*02:03<br>HLA-A*68:02<br>HLA-A*32:01<br>HLA-B*08:01<br>HLA-A*26:01<br>HLA-B*07:02	8	100%	Allergen	Non-toxic
	IAIVMVTIM	1225-1233	1.1339	0.06312	HLA-B*51:01;<br>HLA-B*35:01	2	100%	Non-allergen	Non-toxic
	PYRVVVLSF	507-515	1.0281	0.03138	HLA-A*23:01;<br>HLA-A*24:02	2	100%	Non-allergen	Non-toxic
South Africa	IAIPINFTI	712-720	1.5131	0.27703	HLA-B*51:01;<br>HLA-B*58:01;<br>HLA-B*57:01;<br>HLA-A*02:06;<br>HLA-A*68:02;<br>HLA-B*53:01;<br>HLA-A*32:01;<br>HLA-A*02:01;<br>HLA-A*23:01;<br>HLA-B*35:01;<br>HLA-A*24:02	11	100%	Non-allergen	Non-toxic
	FTISVTTEI	718-726	0.8534	0.04473	HLA-A*68:02;<br>HLA-A*02:06;<br>HLA-A*02:03;<br>HLA-A*02:01;<br>HLA-B*51:01;<br>HLA-A*26:01;<br>HLA-B*58:01;<br>HLA-A*32:01;<br>HLA-B*53:01	9	100%	Non-allergen	Non-toxic
	YQPYRVVVL	505-513	0.5964	0.1409	HLA-B*08:01;<br>HLA-A*02:06;<br>HLA-B*15:01;	8	100%	Non-allergen	Non-toxic

					HLA-A*02:03; HLA-A*02:01; HLA-A*24:02; HLA-B*40:01 HLA-A*23:01				
	WTAGAAAYY	258-266	0.6306	0.15259	HLA-A*26:01; HLA-A*01:01; HLA-A*30:02; HLA-A*68:01; HLA-B*35:01; HLA-B*15:01; HLA-B*58:01	7	100%	Non-allergen	Non-toxic
	YSKHTPINL	204-212	1.0547	0.09845	HLA-B*57:01; HLA-A*30:01; HLA-B*08:01; HLA-B*58:01; HLA-A*68:02; HLA-B*51:01; HLA-A*32:01	7	100%	Non-allergen	Non-toxic
	LPFNDGVYF	84-92	0.5593	0.11767	HLA-B*35:01; HLA-B*53:01; HLA-B*51:01; HLA-B*07:02; HLA-A*26:01	5	100%	Non-allergen	Non-toxic
	GVVFLHVTY	1059-1067	1.4104	0.20837	HLA-B*15:01; HLA-A*30:02; HLA-A*26:01; HLA-B*35:01; HLA-A*32:01	5	100%	Non-allergen	Non-toxic
	GAAAYYVGY	261-269	0.6604	0.09963	HLA-A*30:02; HLA-B*15:01; HLA-B*35:01; HLA-A*26:01; HLA-A*01:01	5	100%	Non-allergen	Non-toxic
	QLTPTWRVY	628-636	1.2119	0.31555	HLA-B*35:01; HLA-B*53:01; HLA-B*51:01; HLA-B*07:02; HLA-A*26:01	5	100%	Non-allergen	Non-toxic
	GVYFASTEK	89-97	0.7112	0.09023	HLA-A*11:01; HLA-A*03:01; HLA-A*68:01; HLA-A*30:01	4	100%	Non-allergen	Non-toxic
	RVVVLSFEL	509-517	0.1918	0.04571	HLA-A*32:01 HLA-A*02:06 HLA-B*57:01 HLA-B*58:01	4	100%	Allergen	Non-toxic
	AEIRASANL	1016-1024	0.7082	0.00689	HLA-B*40:01; HLA-B*44:03; HLA-B*44:02	3	100%	Non-allergen	Non-toxic
	IGAGICASY	666-674	0.6368	0.06201	HLA-A*30:02; HLA-B*15:01; HLA-B*35:01	3	100%	Non-allergen	Non-toxic
	TLADAGFIK	827-835	0.5781	0.28158	HLA-A*11:01; HLA-A*03:01; HLA-A*68:01	3	100%	Non-allergen	Non-toxic

	GQTGKIADY	413-421	1.4019	0.00796	HLA-B*15:01; HLA-A*30:02; HLA-A*26:01	3	100%	Non-allergen	Non-toxic
	VRFPNITNL	327-335	1.1141	0.1748	HLA-A*23:01; HLA-B*08:01; HLA-A*24:02	3	100%	Non-allergen	Non-toxic
	IAIVMVTIM	1225-1233	1.1339	0.06312	HLA-B*51:01; HLA-B*35:01	2	100%	Non-allergen	Non-toxic
	PYRVVVLSF	507-515	1.0281	0.03138	HLA-A*23:01; HLA-A*24:02	2	100%	Non-allergen	Non-toxic
