# Supplementary material for: Comparison of Immunological Profiles of SARS-CoV-2 Variants in the COVID-19 Pandemic Trends: An Immunoinformatics Approach
Source: Antibiotics (Basel). 2021 May 6;10(5):535. doi: 10.3390/antibiotics10050535 (PMC8148159; doi:10.3390/antibiotics10050535)
Supplement: Supplementary file 1 [file antibiotics-10-00535-s001.zip › Supplementary Table S3.pdf]

Table S3 Predicted linear B-cell epitopes of SARS-CoV-2 variants predicted by IEDB-B-cell linear epitope prediction tool. Antigenic, immunogenic, non-allergic/allergic, and non-toxic/toxic B-cell epitopes identified in the S protein of each variant are presented in this table.

Wuhan isolate							
**Sl No**	**Start**	**End**	**Length**	**Peptide**	**VaxiJen score**	**Allergenicity score**	**Toxicity**
1	13	37	25	SQCVNLTTRTQLPPAYTNSFTRGVY	0.5659 (antigen)	Non-allergen	Non-toxic
2	59	81	23	FSNVTWFHAIHVSGTNGTKRFDN	0.6767 (antigen)	Non-allergen	Non-toxic
3	97	98	2	KS	-	Non-allergen	Non-toxic
4	138	154	17	DPFLGVYYHKNNKSWME	0.5821 (antigen)	allergen	Non-toxic
5	177	189	13	MDLEGKQGNFKNL	1.2592 (antigen)	Non-allergen	Non-toxic
6	206	221	16	KHTPINLVRDLPQGFS	0.6403 (antigen)	allergen	Non-toxic
7	250	260	11	TPGDSSSGWTA	0.2473 (non-antigen)	allergen	Non-toxic
8	293	296	4	LDPL	-	Non-allergen	Non-toxic
9	304	322	19	KSFTVEKGIYQTSNFRVQP	0.5729 (antigen)	-	Non-toxic
10	329	363	35	FPNITNLCPFGEVFNATRFASVYAWNRKRISNCVA	0.4466 (non-antigen)	Non-allergen	Non-toxic
11	369	393	25	YNSASFSTFKCYGVSPTKLNDLCFT	1.4031 (antigen)	Non-allergen	Non-toxic
12	404	426	23	GDEVRQIAPGQTGKIADYNYKLP	1.1017 (antigen)	allergen	Non-toxic
13	440	501	62	NLDSKVGGNYNYLYRLFRKSNLKPFERDISTEIYQAGSTPCNGVEGFNCYFPLQSYGFQPTN	0.395 (non-antigen)	Non-allergen	Non-toxic
14	516	536	21	ELLHAPATVCGPKKSTNLVKN	0.00029 (non-antigen)	allergen	Non-toxic
15	555	562	8	SNKKFLPF	1.3952 (antigen	Non-allergen	Non-toxic

					)	n	
16	580	583	4	QTLE	Non-antigen	-	Non-toxic
17	602	606	5	TNTSN	Non-antigen	allergen	Non-toxic
18	616	632	17	NCTEVPVAIHADQLTPT	0.3989 (non-antigen)	allergen	Non-toxic
19	634	644	11	RVYSTGSNVFQ	-0.1000 (non-antigen)	allergen	Non-toxic
20	656	666	11	VNNSYECDIPI	0.6124 (antigen)	allergen	Non-toxic
21	672	690	19	ASYQTQTNSPRRARSVASQ	0.2556 (non-antigen)	allergen	Non-toxic
22	695	710	16	YTMSLGAENSVAYSNN	0.6434 (antigen)	Non-allergen	Non-toxic
23	748	748	1	E	-	-	-
24	773	779	7	EQDKNTQ	0.1017 (non-antigen)	Non-allergen	Non-toxic
25	786	800	15	KQIYKTPPIKDFGGF	-0.3896 (non-antigen)	Non-allergen	Non-toxic
27	807	814	8	PDPSKPSK	0.0621 (non-antigen)	allergen	Non-toxic
28	828	842	15	LADAGFIKQYGDCLG	0.2071 (non-antigen)	Non-allergen	Non-toxic
29	988	992	5	EAEVQ	-	-	Non-toxic
30	1035	1043	9	GQSKRVDFC	1.7790 (antigen)	allergen	Non-toxic
31	1107	1118	12	RNFYEPQIITTD	0.3529 (non-antigen)	allergen	Non-toxic
32	1133	1172	40	VNNTVYDPLQPELDSFKEELDKYFKNHTSPDVDLGDISGI	0.161 (non-antigen)	allergen	Non-toxic
33	1203	1206	4	LGKY	-	non-allergen	Non-toxic
34	1252	1267	16	SCCKFDEDDSEPVLKG	0.4347 (non-antigen)	Non-allergen	toxic
35	1269	1269	1	K	-	-	-
**England isolate**							

36	13	37	25	SQCVNLTTRTQLPPAYTNSFTRGVY	0.686 (antigen)	Non-allergen	Non-toxic
37	59	81	23	FSNVTWFHAIHVSGTNGTKRFDN	0.6767 (antigen_	Non-allergen	Non-toxic
38	96	100	5	EKSNI	-	allergen	Non-toxic
39	138	156	19	DPFLGVYYHKNNKSWMESE	0.5959 (antigen)	Non-allergen	Non-toxic
40	176	189	14	LMDLEGKQGNFKNL	1.1506 (antigen)	Non-allergen	Non-toxic
41	208	223	16	TPINLVRDLPQGFSAL	0.5912 (antigen)	allergen	Non-toxic
42	249	260	12	LTPGDSSSGWTA	0.689 (antigen)	allergen	Non-toxic
43	294	294	1	D	-	-	-
44	296	296	1	L	-	-	-
45	306	322	17	FTVEKGIYQTSNFRVQP	0.6549 (antigen)	allergen	Non-toxic
46	329	356	28	FPNITNLCPFGEVFNATRFASVYAWNRK	0.5517 (antigen)	Non-allergen	Non-toxic
47	367	395	29	VLYNSASFSTFKCYGVSPTKLNDLCFTNV	1.1825 (antigen)	Non-allergen	Non-toxic
48	403	421	19	RGDEVRQIAPGQTGKIADY	0.9522 (antigen)	allergen	Non-toxic
49	423	427	5	YKLPD	-	Non-allergen	Non-toxic
50	439	450	12	NNLDSKVGGNYN	0.7538 (antigen)	Non-allergen	Non-toxic
51	458	495	38	KSNLKPFERDISTEIYQAGSTPCNGVEGFNCYFPLQSY	0.3212 (non-antigen)	allergen	Non-toxic
52	516	535	20	ELLHAPATVCGPKKSTNLVK	0.0205 (non-antigen)	Non-allergen	Non-toxic
53	557	561	5	KKFLP	-	allergen	Non-toxic
54	580	583	4	QTLE	-	allergen	Non-toxic
55	603	606	4	NTSN	-	Non-allergen	Non-toxic
56	616	644	29	NCTEVPVAIHADQLTPTWRVYSTGSNVFQ	0.3197 (non-	allergen	Non-toxic

					antigen)		
57	656	667	12	VNNSYECDIPIG	0.9148 (antigen)	Non-allergen	Non-toxic
58	671	690	20	CASYQTQTNSPRRARSVASQ	0.2665 (non-antigen)	allergen	Non-toxic
59	695	710	16	YTMSLGAENSVAYSNN	0.6434 (antigen)	Non-allergen	Non-toxic
60	748	748	1	E	-	Non-allergen	-
61	775	779	5	DKNTQ	-	Non-allergen	Non-toxic
62	785	800	16	VKQIYKTPPIKDFGGF	-0.2341 (non-antigen)	allergen	Non-toxic
63	806	815	10	LPDPSKPSKR	0.2641 (non-antigen)	Non-allergen	Non-toxic
64	828	843	16	LADAGFIKQYGDCLGD	0.0965 (non-antigen)	Non-allergen	Non-toxic
65	887	888	2	TF	-	allergen	Non-toxic
66	987	993	7	VEAEVQI	0.8205 (antigen)	Non-allergen	Non-toxic
67	1035	1043	9	GQSKRVDFC	1.779 (antigen)	allergen	Non-toxic
68	1109	1118	10	FYEPQIITTD	0.4179 (non-antigen)	Non-allergen	Non-toxic
69	1133	1172	40	VNNTVYDPLQPELDSFKEELDKYFKNHTSPDVDLGDISGI	0.1613 (non-antigen)	allergen	Non-toxic
70	1203	1207	5	LGKYE	-	Non-allergen	Non-toxic
71	1253	1269	17	CCKFDEDDSEPVLKGVK	0.6515 (antigen)	Non-allergen	toxic
**USA isolate**							
72	13	37	25	SQCVNLTTRTQLPPAYTNSFTRGVY	0.6860 (antigen)	Non-allergen	Non-toxic
73	59	81	23	FSNVTWFHAIHVSGTNGTKRFDN	0.6767 (antigen)	Non-allergen	Non-toxic
74	97	98	2	KS	-	Allergen	Non-toxic
75	138	154	17	DPFLGVYYHKNNKSWME	0.5821	Allerge	Non-

					(antigen)	n	toxic
76	177	189	13	MDLEGKQGNFKNL	1.2592 (antigen)	Non-allergen	Non-toxic
77	206	221	16	KHTPINLVRDLPQGFS	0.6403 (antigen)	Allergen	Non-toxic
78	250	260	11	TPGDSSSGWTA	0.2473 (antigen)	allergen	Non-toxic
79	293	296	4	LDPL	-	allergen	Non-toxic
80	304	322	19	KSFTVEKGIYQTSNFRVQP	0.5729 (antigen)	allergen	Non-toxic
81	329	363	35	FPNITNLCPFGEVFNATRFASVYAWNRKRISNCVA	0.4466 (non-antigen)	Non-allergen	Non-toxic
82	369	393	25	YNSASFSTFKCYGVSPTKLNDLCFT	1.4031 (antigen)	Non-allergen	Non-toxic
83	404	426	23	GDEVRQIAPGQTGKIADYNYKLP	1.1017 (antigen)	allergen	Non-toxic
84	440	501	62	NLDSKVGGNYNYLYRLFRKSNLKPFERDISTEIYQAGSTPCNGVEGFNCYFPLQSYGFQPTN	0.3951 (non-antigen)	Non-allergen	Non-toxic
85	516	536	21	ELLHAPATVCGPKKSTNLVKN	0.0029 (non-antigen)	allergen	Non-toxic
86	555	562	8	SNKKFLPF	1.3952 (antigen)	Non-allergen	Non-toxic
87	580	583	4	QTLE	-	allergen	Non-toxic
88	602	606	5	TNTSN	-	allergen	Non-toxic
89	617	632	16	CTEVPVAIHADQLTPT	0.2794 (non-antigen)	Non-allergen	Non-toxic
90	635	643	9	VYSTGSNVF	-0.3099 (non-antigen)	allergen	Non-toxic
91	656	666	11	VNNSYECDIPI	0.6124 (antigen)	allergen	Non-toxic
92	672	690	19	ASYQTQTNSPRRARSVASQ	0.2556 (non-antigen)	allergen	Non-toxic
93	695	710	16	YTMSLGAENSVAYSNN	0.6434 (antigen)	Non-allergen	Non-toxic
94	748	748	1	E	-	Non-allergen	Non-toxic

95	773	779	7	EQDKNTQ	0.1017 (non-antigen)	Non-allergen	Non-toxic
96	786	800	15	KQIYKTPPIKDFGGF	-0.3896 (non-antigen)	Non-allergen	Non-toxic
97	807	814	8	PDPSKPSK	0.0621 (non-antigen)	allergen	Non-toxic
98	828	842	15	LADAGFIKQYGDCLG	0.2071 (non-antigen)	Non-allergen	Non-toxic
99	988	992	5	EAEVQ	-	allergen	Non-toxic
100	1035	1043	9	GQSKRVDFC	1.779 (antigen)	allergen	Non-toxic
101	1107	1118	12	RNFYEPQIITTD	0.3529 (antigen)	allergen	Non-toxic
102	1133	1172	40	VNNTVYDPLQPELDSFKEELDKYFKNHTSPDVDLGDISGI	0.1613 (non-antigen)	allergen	Non-toxic
103	1203	1206	4	LGKY	-	Non-allergen	Non-toxic
104	1252	1267	16	SCCKFDEDDSEPVLKG	0.4347 (non-antigen)	Non-allergen	Toxic
105	1269	1269	1	K	-	Non-allergen	-
**Indian isolate**							
106	13	37	25	SQCVNLTTRTQLPPAYTNSFTRGVY	0.6860 (antigen)	Non-allergen	Non-toxic
107	60	62	3	SNV	-	Non-allergen	Non-toxic
108	68	82	15	GAGSGTNGTKRFDNP	0.461 (non-antigen)	Non-allergen	Non-toxic
109	84	84	1	L	-	Non-allergen	Non-toxic
110	97	98	2	KS	-		Non-toxic
111	138	156	19	DPFLGVYYHKNNKSWMESE	0.5959 (antigen)	Non-allergen	Non-toxic
112	178	189	12	DLEGKQGNFKNL	1.1816 (antigen)	Non-allergen	Non-toxic
113	208	223	16	TPINLVRDLPQGFSAL	0.5912 (antigen)	Allergen	Non-toxic
114	249	260	12	LTPGDSSSGWTA	0.6890 (antige	Non-allergen	Non-toxic

					n)		
115	294	294	1	D	-	Non-allergen	Non-toxic
116	296	296	1	L	-	Non-allergen	Non-toxic
117	306	323	18	FTVEKGIYQTSNFRVQPT	0.7469 (antigen)	allergen	Non-toxic
118	329	356	28	FPNITNLCPFGEVFNATRFASVYAWNRK	0.5517 (antigen)	allergen	Non-toxic
119	364	393	30	DYSVLYNSASFSTFKCYGVSPTKLNDLCFT	1.1777 (antigen)	Non-allergen	Non-toxic
120	401	421	21	VIRGDEVRQIAPGQTGKIADY	0.7943 (antigen)	Allergen	Non-toxic
121	423	427	5	YKLPD	-	Non-allergen	Non-toxic
122	439	451	13	NNLDSKVGGNYNY	0.9437 (non-antigen)	Allergen	Non-toxic
123	459	467	9	SNLKPFERD	0.8527 (antigen)	Non-allergen	Non-toxic
124	471	502	32	EIYQAGSTPCNGVEGFNCYFPLQSYGFQPTYG	0.4047 (non-antigen)	Allergen	Non-toxic
125	516	534	19	ELLHAPATVCGPKKSTNLV	0.1474 (non-antigen)	Allergen	Non-toxic
126	555	562	8	SNKKFLPF	1.3952 (antigen)	Allergen	Non-toxic
127	581	582	2	TL	-	-	Non-toxic
128	603	605	3	NTS	-	-	Non-toxic
129	617	645	29	CTEVPVAIHADQLTPTWRVYSTGSNVFQT	0.2795 (non-antigen)	Non-allergen	Non-toxic
130	656	665	10	VNNSYECDIP	0.5327 (antigen)	Non-allergen	Non-toxic
131	672	690	19	ASYQTQTNSHRRARSVASQ	0.4584 (non-antigen)	Non-allergen	Non-toxic
132	695	710	16	YTMSLGAENSVAYSNN	0.6434 (antigen)	Non-allergen	Non-toxic
133	748	748	1	E	-	Non-allergen	Non-toxic

134	773	779	7	EQDKNTQ	0.1017 (non-antigen)	Allergen	Non-toxic
135	786	800	15	KQIYKTPPIKDFGGF	-0.3896 (non-antigen)	Non-allergen	Non-toxic
136	807	814	8	PDPSKPSK	0.0621 (non-antigen)	Allergen	Non-toxic
137	828	843	16	LADAGFIKQYGDCLGD	0.0965 (non-antigen)	Allergen	Non-toxic
138	988	992	5	EAEVQ	-	Non-allergen	Non-toxic
139	1035	1043	9	GQSKRVDFC	1.7790 (antigen)	Allergen	Non-toxic
140	1107	1118	12	RNFYEPQIITTH	0.3349 (non-antigen)	Allergen	Non-toxic
141	1133	1172	40	VNNTVYDPLQPELDSFKEELDKYFKNHTSPDVDLGDISGI	0.1613 (non-antigen)	Non-allergen	Non-toxic
142	1203	1206	4	LGKY	-	Allergen	Non-toxic
143	1252	1269	18	SCCKFDEDDSEPVLKGVK	0.5409 (antigen)	Non-allergen	Toxic
**South African isolate**							
144	13	37	25	SQCVNLTTRTQLPPAYTNSFTRGVY	0.6860 (antigen)	Non-allergen	Non-toxic
145	59	81	23	FSNVTWFHAIHVSGTNGTKRFDN	0.6767 (antigen)	Non-allergen	Non-toxic
146	97	98	2	KS	-	Allergen	Non-toxic
147	138	154	17	DPFLGVYYHKNNKSWME	0.5821 (antigen)	Non-allergen	Non-toxic
148	177	189	13	MDLEGKQGNFKNL	1.2592 (antigen)	Non-allergen	Non-toxic
149	206	221	16	KHTPINLVRDLPQGFS	0.6403 (antigen)	Non-allergen	Non-toxic
150	250	260	11	TPGDSSSGWTA	0.2473 (non-antigen)	Non-allergen	Non-toxic
151	293	296	4	LDPL	-	Non-allergen	Non-toxic

152	304	322	19	KSFTVEKGIYQTSNFRVQP	0.5729 (antigen)	Allergen	Non-toxic
153	329	363	35	FPNITNLCPFGEVFNATRFASVYAWNRKRISNCVA	0.4466 (non-antigen)	Allergen	Non-toxic
154	369	393	25	YNSASFSTFKCYGVSPTKLNDLCFT	1.4031 (antigen)	Non-allergen	Non-toxic
155	404	426	23	GDEVRQIAPGQTGKIADYNYKLP	1.1017 (antigen)	Non-allergen	Non-toxic
156	440	501	62	NLDSKVGGNYNYLYRLFRKSNLKPFERDISTEIYQAGSTPCNGVEGFNCYFPLQSYGFQPTN	0.3951 (non-antigen)	Allergen	Non-toxic
157	516	536	21	ELLHAPATVCGPKKSTNLVKN	0.0029 (non-antigen)	Allergen	Non-toxic
158	555	562	8	SNKKFLPF	1.3952 (antigen)	Allergen	Non-toxic
159	580	583	4	QTLE	-	Non-allergen	Non-toxic
160	602	606	5	TNTSN	-	Non-allergen	Non-toxic
161	617	632	16	CTEVPVAIHADQLTPT	0.2794 (non-antigen)	Non-allergen	Non-toxic
162	635	643	9	VYSTGSNVF	-0.3099 (non-antigen)	Non-allergen	Non-toxic
163	656	666	11	VNNSYECDIPI	0.6124 (antigen)	Non-allergen	Non-toxic
164	672	690	19	ASYQTQTNSPRRARSVASQ	0.2556 (non-antigen)	Non-allergen	Non-toxic
165	695	710	16	YTMSLGAENSVAYSNN	0.6434 (antigen)	Non-allergen	Non-toxic
166	748	748	1	E	-	Non-allergen	Non-toxic
167	773	779	7	EQDKNTQ	0.1017 (non-antigen)	Allergen	Non-toxic
168	786	800	15	KQIYKTPPIKDFGGF	0.3896	Non-	Non-

					(non-antigen)	allergen	toxic
169	807	814	8	PDPSKPSK	0.0621 (non-antigen)	Allergen	Non-toxic
170	828	842	15	LADAGFIKQYGDCLG	0.2071 (non-antigen)	Allergen	Non-toxic
171	988	992	5	EAEVQ	-	Non-allergen	Non-toxic
172	1035	1043	9	GQSKRVDFC	1.7790 (antigen)	Allergen	Non-toxic
173	1107	118	12	RNFYEPQIITTD	0.3529 (non-antigen)	Allergen	Non-toxic
174	1133	1172	40	VNNTVYDPLQPELDSFKEELDKYFKNHTSPDVDLGDISGI	0.1613 (non-antigen)	Non-allergen	Non-toxic
175	1203	1206	4	LGKY	-	Allergen	Toxic
176	1252	1267	16	SCCKFDEDDSEPVLKG	0.4347 (non-antigen)	Allergen	Toxic
177	1269	1269	1	K	-	Non-allergen	Non-toxic
